# Integrating Spatial and Single‐Nucleus Transcriptomic Data to Assess the Effects of Intrauterine Hyperglycemia on Fetal Pancreatic Development

**DOI:** 10.1002/advs.202411126

**Published:** 2025-05-19

**Authors:** Yu Deng, Shuting Wan, Zan Yuan, Huixia Yang

**Affiliations:** ^1^ Department of Obstetrics and Gynecology Peking University First Hospital No. 8 Xishiku Street Beijing 100034 China; ^2^ Beijing Key Laboratory of Maternal Fetal Medicine of Gestational Diabetes Mellitus Beijing 100034 China; ^3^ Annoroad Gene Technology (Beijing) Co., Ltd Beijing 100176 China; ^4^ Agricultural Bioinformatics Key Laboratory of Hubei Province Hubei Engineering Technology Research Center of Agricultural Big Data College of Informatics Huazhong Agricultural University Wuhan 430070 China

**Keywords:** fetal pancreas, pregestational diabetes mellitus, single‐nucleus RNA sequencing, spatial transcriptomics

## Abstract

Maternal pregestational diabetes mellitus (PGDM) can lead to adverse fetal outcomes, including lasting impacts on pancreatic development. However, the specific impacts of maternal PGDM on cellular functions and intercellular communication within the fetal pancreas remain poorly understood. Here, single‐nucleus RNA sequencing and spatial transcriptomics (ST) are employed to investigate cellular responses and spatial changes in the fetal pancreas (E16.5 and E18.5) under maternal PGDM conditions. The findings reveal significant cellular heterogeneity among acinar and beta cells, along with pronounced metabolic stress responses. More importantly, decreased insulin secretion is observed and accompanied by the compensatory increase of Pdx1, Nkx6.2, and Mafa, and substantial alterations in cell‐cell communication across multiple cell types. ST analysis further highlights enhanced spatial enrichment in cellular niches exposed to maternal PGDM. These findings provide valuable insights into the molecular mechanisms underlying fetal pancreatic response to maternal PGDM and offer a detailed spatiotemporal perspective on these processes.

## Graphical abstract

Maternal pregestational diabetes mellitus (PGDM) induced metabolic stress in fetal mice pancreas, alongside significantly decreased insulin secretion, altered cell‐cell communication, and distinct changes in cellular niches.

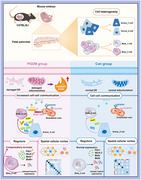

## 1 Introduction

The prevalence of diabetes mellitus has steadily increased over the past few decades.^[^ ^1^ ^]^ Pregestational diabetes mellitus (PGDM) is a significant risk factor for complications during pregnancy and childbirth.^[^ ^1^, ^2^ ^]^ Although PGDM occurs less frequently in pregnant women (0.92%–1.18%), it is associated with a high risk of short‐ and long‐term adverse fetal and maternal outcomes, such as metabolic dysfunction and hypertension.^[^ ^1^, ^2^, ^3^ ^]^ Therefore, more in‐depth research into PGDM is urgently needed as a critical public health issue.

The pancreas plays a vital role in regulating glucose homeostasis, and it must adapt to the metabolic stress imposed by pregnancy.^[^ ^4^ ^]^ This process involves hypertrophy and hyperplasia of pancreatic endocrine cells.^[^ ^5^ ^]^ Recent clinical studies^[^ ^6^ ^]^ have shown that women with elevated blood glucose levels during pregnancy experience worsened insulin sensitivity, potentially disrupting glucose‐insulin homeostasis in their offspring. Animal experiments have further indicated that fetal rats exposed to hyperglycemia in late‐pregnancy^[^ ^7^ ^]^ or throughout gestation^[^ ^8^ ^]^ exhibit decreased insulin sensitivity and impaired beta‐cell function compared to controls after birth. Fetal development is a critical period for pancreatic growth, with lasting effects on the individual's metabolic health throughout life.^[^ ^9^ ^]^ However, prior studies on the offspring of mothers with PGDM have largely focused on pancreatic function in adulthood, neglecting the effects during fetal development. Furthermore, these studies have lacked the single‐cell and spatial resolution to elucidate the specific effects of maternal hyperglycemia on the fetal pancreas.

With the advent of technologies such as single‐cell RNA sequencing,^[^ ^9^, ^10^ ^]^ single‐nucleus RNA sequencing (snRNA‐seq),^[^ ^11^ ^]^ and spatial transcriptomics (ST),^[^ ^12^ ^]^ a deeper understanding of pancreas development in humans and mice has been achieved. Although animal models have limitations, they remain indispensable for fetal pancreatic research due to the ethical and practical challenges of conducting clinical trials in pregnant women. A recent study^[^ ^10^ ^]^ established a mouse islet atlas using diverse datasets, but little is known about the complex cellular responses to metabolic stress or intercellular communication changes within the fetal pancreas in the context of maternal PGDM.

In this study, we performed snRNA‐seq and ST analysis to explore the effects of maternal PGDM on the fetal pancreas (E16.5 and E18.5). Our multi‐omics approach provided the following key findings: (1) we identified functional and metabolic heterogeneity among beta‐cells and acinar cells in both PGDM‐exposed group and control fetal mice; (2) snRNA‐seq revealed pronounced cellular responses to metabolic stress in the PGDM group, with transmission electron microscopy (TEM) confirming significant swelling of ribosomal and mitochondrial structures; (3) we observed decreased insulin secretion accompanied by the compensatory increase of Pdx1, Nkx6.2 and Mafa, and substantial alterations in cell‐cell communication across multiple cell types in the PGDM group; and (4) ST analysis demonstrated increased spatial enrichment in various cellular niches within the fetal pancreas exposed to maternal PGDM. These findings suggest that maternal PGDM profoundly impacts fetal pancreatic development and may provide valuable insights for preventing adverse metabolic health outcomes in offspring.

## 2 Results

### 2.1 Global Single‐Nucleus Transcriptome Map of the Fetal Pancreas (E16.5 and E18.5)

The data shown in Figure S1 (Supporting Information) demonstrated that the moderate‐to‐severe mouse model of PGDM was successfully constructed (Figure S1, Supporting Information).^[^ ^2b^ ^]^ There was a significant decrease in the body weights of fetal mice and the numbers of live fetuses per litter in the PGDM group compared with the control group at E12.5, E14.5, E16.5, and E18.5 (Figure S1, Supporting Information).

**Figure 1:**
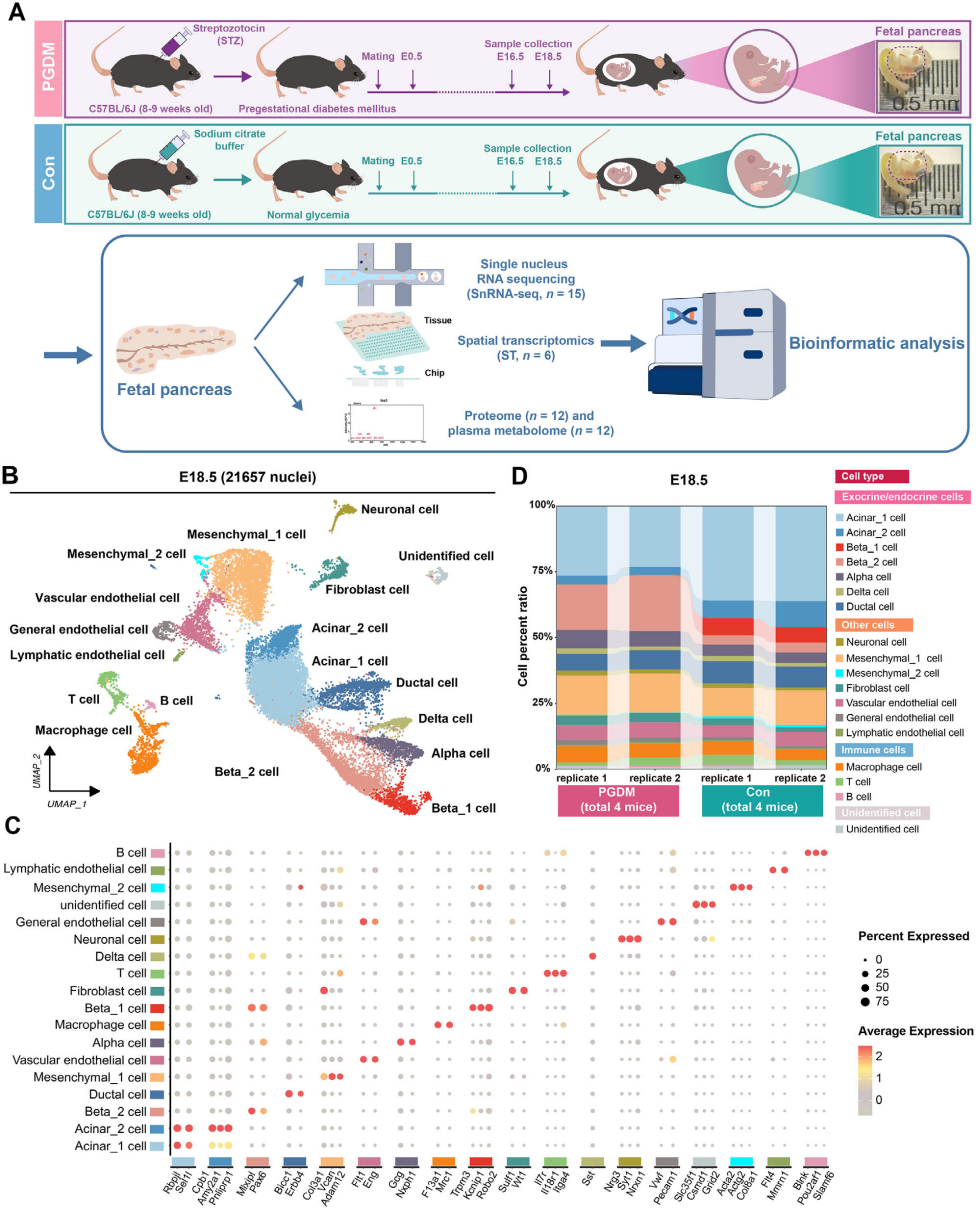
Global single‐nucleus transcriptome map of fetal pancreas (E18.5). A) Schematic for the experimental design and workflow for data processing steps. B) Uniform manifold approximation and projection (UMAP) representation of the fetal pancreas (E18.5). C) Dotplot showing the expression levels of specific marker genes in each cell cluster at E18.5. D) Bar graph showing the cluster composition of the PGDM and control groups at E18.5 in this study. Abbreviations: E0.5, embryonic day E0.5; E16.5, embryonic day E16.5; E18.5, embryonic day E18.5.

To achieve high‐resolution molecular profiling of fetal pancreatic cells in response to maternal PGDM, we conducted snRNA‐seq on fetal pancreas from PGDM and control groups (**Figure** **1**A). After quality control and filtration, 38237 single nuclei (E16.5) and 21657 single nuclei (E18.5) were included in the analysis (Figures S2A–D and S3, Supporting Information).

We identified 17 major cell types, using E18.5 snRNA‐seq data, based on gene expression markers: acinar_1 cells (*Rbpjl* and *Sel1l*), acinar_2 cells (*Cpb1*, *Amy2a1* and *Pnliprp1*), beta_1 cells (*Trpm3*, *Kcnip1*, and *Robo2*), beta_2 cells (*Mlxipl* and *Pax6*), alpha‐cells (*Gcg* and *Nxph1*), delta cells (*Sst*), ductal cells (*Bicc1* and *Erbb4*), mesenchymal _1 cells (*Col3a1*, *Vcan*, and *Adam12*), mesenchymal_2 cells (*Acta2*, *Actg2*, and *Col8a1*), neuronal cells (*Nrg3*, *Syt1*, and *Nrxn1*), vascular endothelial cells (*Flt1* and *Eng*), general endothelial cells (*Vwf* and *Pecam1*), lymphatic endothelial cells (*Flt4* and *Mmrn1*), macrophages (*F13a1* and *Mrc1*), T cells (*Il7r*, *Il18r1*, and *Itga4*), B cells (*Blnk*, *Pou2af1*, and *Slamf6*), and fibroblasts (*Sulf1* and *Wt1*) (Figure 1B, C, and Table S1, Supporting Information). We also identified 16 major cell types using E16.5 snRNA‐seq data (Figure S3 and Table S2, Supporting Information), and we found major cell types at different time points (E16.5 and E18.5) were consistent.

Next, we investigated the potential developmental connectivity among acinar cells, beta cells, and ductal cells at E16.5 and E18.5, with partition‐based graph abstraction (PAGA). The PAGA analysis represented that the acinar cells were highly connected to beta cells and ductal cells (Figure S4A, Supporting Information). Subsequently, we performed pseudotime analysis with the Monocle2 R package to better understand the developmental relationship among acinar and beta cell populations. We found that acinar_2 cells (E16.5) and acinar_3 cells (E16.5) appeared first, followed by acinar_1 cells (E16.5), then acinar_2 cells (E18.5), and finally acinar_1 cell (E18.5); beta_2 cells (E16.5) appeared first, followed by beta_1 cell (E16.5), then beta_2 cell (E18.5), and finally beta_1 cell (E18.5) (Figure S4B, Supporting Information).

In addition, we investigated the heterogeneity of acinar_1 cells, acinar_2 cells, beta_1 cells, and beta_2 cells by performing cluster composition analysis. This analysis revealed substantial differences in the composition of these cell types between the PGDM (E18.5) and control groups (E18.5), while the composition of other clusters remained relatively unaffected (Figure 1D). Notably, we observed significant metabolic heterogeneity within the acinar_1 cells, acinar_2 cells, beta_1 cells, and beta_2 cells at E18.5 (Figure S5, Supporting Information). Metabolic pathway analysis revealed differential expression of several pathways in these cell types, including glycolipid metabolism, glycolysis/gluconeogenesis, nitrogen metabolism, phenylalanine metabolism, steroid biosynthesis, sulfur metabolism, and taurine and hypotaurine metabolism (Figure S6, Supporting Information).

Further analysis showed that glycolysis/gluconeogenesis‐related pathways were significantly up‐regulated in multiple cell clusters, particularly acinar_1 cells, acinar_2 cells, beta_1 cells, and beta_2 cells, in the PGDM group (E18.5) compared to the control group (E18.5) (Figure S7, Supporting Information). This observation aligned with previous findings in diabetic mice (βV59 M), which exhibited high expression of glycolysis‐related genes despite reduced glucose utilization.^[^ ^13^ ^]^ In summary, our data revealed substantial heterogeneity in acinar cells and beta cells between the PGDM and control groups, particularly in their metabolic profiles.

### 2.2 Pronounced Cellular Response to Metabolic Stress Caused by Maternal PGDM in the Fetal Pancreas at E16.5 and E18.5

**Figure 2:**
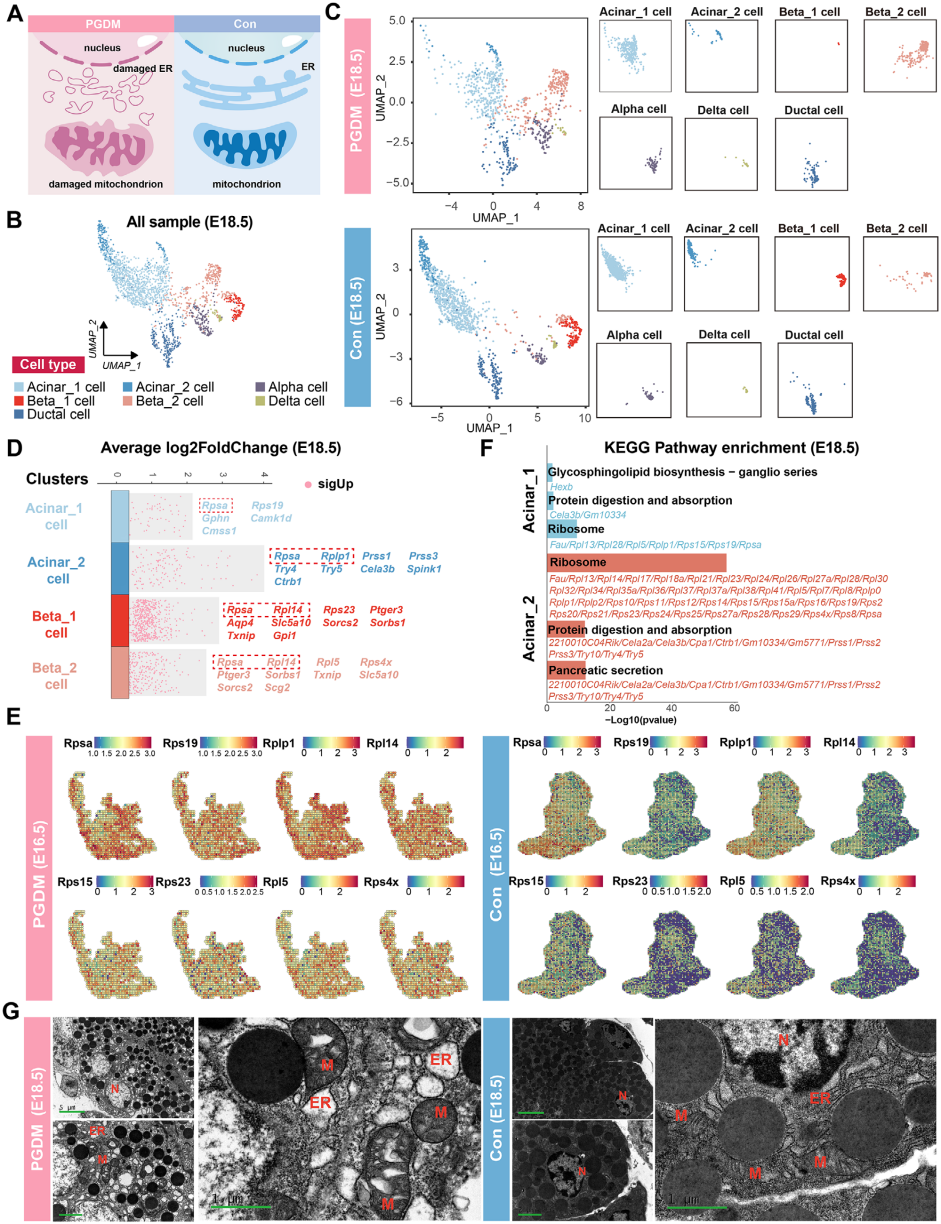
Maternal PGDM caused increased cellular response to stress in the fetal pancreas at E16.5 and E18.5. A) Schematic diagram showing metabolic stress in the PGDM (left) and control (right) groups. B) UMAP plot showing seven cell populations in the PGDM (E18.5) and control (E18.5) groups. C) UMAP plot showing seven cell populations in the PGDM (E18.5, upper panel) and control (E18.5, lower panel) groups. D) Depiction of up‐regulated differentially expressed genes (DEGs) for the acinar_1 cell, acinar_2 cell, beta_1 cell, and beta_2 cell populations in the PGDM (E18.5) group, compared to the control (E18.5) group. Red dots represented up‐regulated DEGs. E) ST data based on spots (bin 50, 50 × 50 nanoballs aggregated) showing gene expression levels of *Rpsa*, *Rps19*, *Rplp1*, and *Rpl14* in the PGDM (E16.5) and control (E16.5) groups. F) Top five enriched KEGG pathways of up‐regulated DEGs in the acinar_1 and acinar_2 cell populations in the PGDM (E18.5) group, compared to the control (E18.5) group. G) Electron microscopy revealed impairments in the mitochondria and endoplasmic reticulum in the PGDM (E18.5) group compared to the control (E18.5) group. Abbreviations: ER, endoplasmic reticulum; M, mitochondria; N, nucleus; ST, spatial transcriptomics.

A previous study^[^ ^14^ ^]^ indicated that high glucose induced severe metabolic disorders in pancreatic cells. Therefore, we further explored the cellular response to metabolic stress in the fetal pancreas (**Figure** **2**A). To accurately characterize the cell populations of acinar_1 cell, acinar_2 cell, beta_1 cell, and beta_2 cell, we first presented seven major cell types from the E18.5 samples using uniform manifold approximation and projection (UMAP) (Figure 2B). A split‐by‐group UMAP clearly demonstrated that beta_2 cells were enriched in the PGDM (E18.5) group, while acinar_2 cells and beta_1 cells were more prevalent in the control (E18.5) group (Figure 2C).

Next, we identified differentially expressed genes (DEGs) that were up‐regulated between the PGDM (E18.5) and control (E18.5) groups across the four clusters mentioned above (Figure 2D). For example, the analysis of DEGs from the acinar_1 cell population revealed that genes such as *Rpsa*, *Rps19*, *Gphn*, *Camk1d*, and *Cmss1* were significantly up‐regulated in the PGDM (E18.5) group compared to the control (E18.5) group (Figure 2D). Similarly, in the acinar_2 cell population, genes including *Rpsa*, *Rplp1*, *Prss1*, *Prss3*, *Try4*, *Try5*, *Cela3b*, *Spink1*, and *Ctrb1* were expressed at significantly higher levels in the PGDM (E18.5) group compared to controls (Figure 2D). Analysis of DEGs from the beta_1 cell population demonstrated that the PGDM (E18.5) group had higher expression levels of *Rpsa*, *Rpl14*, *Rps23*, *Ptger3*, *Aqp4*, *Slc5a10*, *Sorcs2*, *Sorbs1*, *Txnip*, and *Gpi1* than the control (E18.5) group (Figure 2D). Similarly, in the beta_2 cell population, genes such as *Rpsa*, *Rpl14*, *Rpl5*, *Rps4x*, *Ptger3*, *Sorbs1*, *Txnip*, *Slc5a10*, *Sorcs2*, and *Scg2* were significantly up‐regulated in the PGDM (E18.5) group compared to controls (Figure 2D).

Interestingly, many of the significantly up‐regulated DEGs, including *Rpsa* and *Rpl14*, were shared across multiple cell clusters (Figure S8A, Supporting Information). These genes were primarily associated with ribosomal complexes (Figure 2D). Protein–protein interaction analysis revealed a tightly connected network among these DEGs (Figure S8B, Supporting Information). In addition, ST results demonstrated notable differences in the expression of *Rpsa*, *Rps19*, *Rplp1*, and *Rpl14* between the PGDM (E16.5) and control (E16.5) groups (Figure 2D). These data provided support for the hypothesis that ribosomal genes might be affected by maternal PGDM at both E16.5 and E18.5. However, additional studies were necessary to further substantiate this perspective.

To further elucidate the biological functions of these DEGs, we performed gene ontology (GO) enrichment analysis and kyoto encyclopedia of genes and genomes (KEGG) pathway analysis on the up‐regulated DEGs in the PGDM (E16.5 and E18.5) group compared to controls (E16.5 and E18.5). The GO enrichment analysis of acinar cells and beta cells at E18.5 highlighted the significant enrichment of genes involved in ribosome biogenesis, ribosomal subunit assembly, cytoplasmic translation, glucose 6−phosphate metabolic process, and glycolytic process through fructose‐6‐phosphate (Figure S9, Supporting Information). The KEGG analysis at E18.5 suggested that significantly up‐regulated signaling pathways related to ribosome and protein digestion and absorption in the acinar_1 and acinar_2 cell populations (Figure 2F) and signaling pathways associated with ribosome in beta_1 cell and beta_2 cell populations (Figure S10, Supporting Information). Similarly, results of GO enrichment analysis of acinar cells and beta cells at E16.5 showed that most of the significantly enriched terms were the regulation of peptidase activity and the regulation of morphogenesis of a branching structure (Figures S11 and S12, Supporting Information). The top significantly enriched KEGG pathways of acinar cells and beta cells at E16.5 were protein digestion and absorption and pancreatic secretion (Figure S13, Supporting Information). Together, the results of GO and KEGG analysis at E16.5 and E18.5 suggested that the up‐regulated signaling pathways in acinar cells and beta cells were correlated with cellular response to metabolic stress.

Secondary validation using TEM confirmed the presence of dilated cisternae in the endoplasmic reticulum, swollen mitochondria, and decreased mitochondrial cristae in the acinar cells of the PGDM (E18.5) group compared to controls (E18.5) (Figure 2G). In conclusion, we observed that the cellular response to metabolic stress was significantly affected in the fetal pancreas of the PGDM group at both E16.5 and E18.5 compared to controls.

### 2.3 The Impairment of Beta Cell Caused by Maternal PGDM in the Fetal Pancreas at E16.5 and E18.5

**Figure 3:**
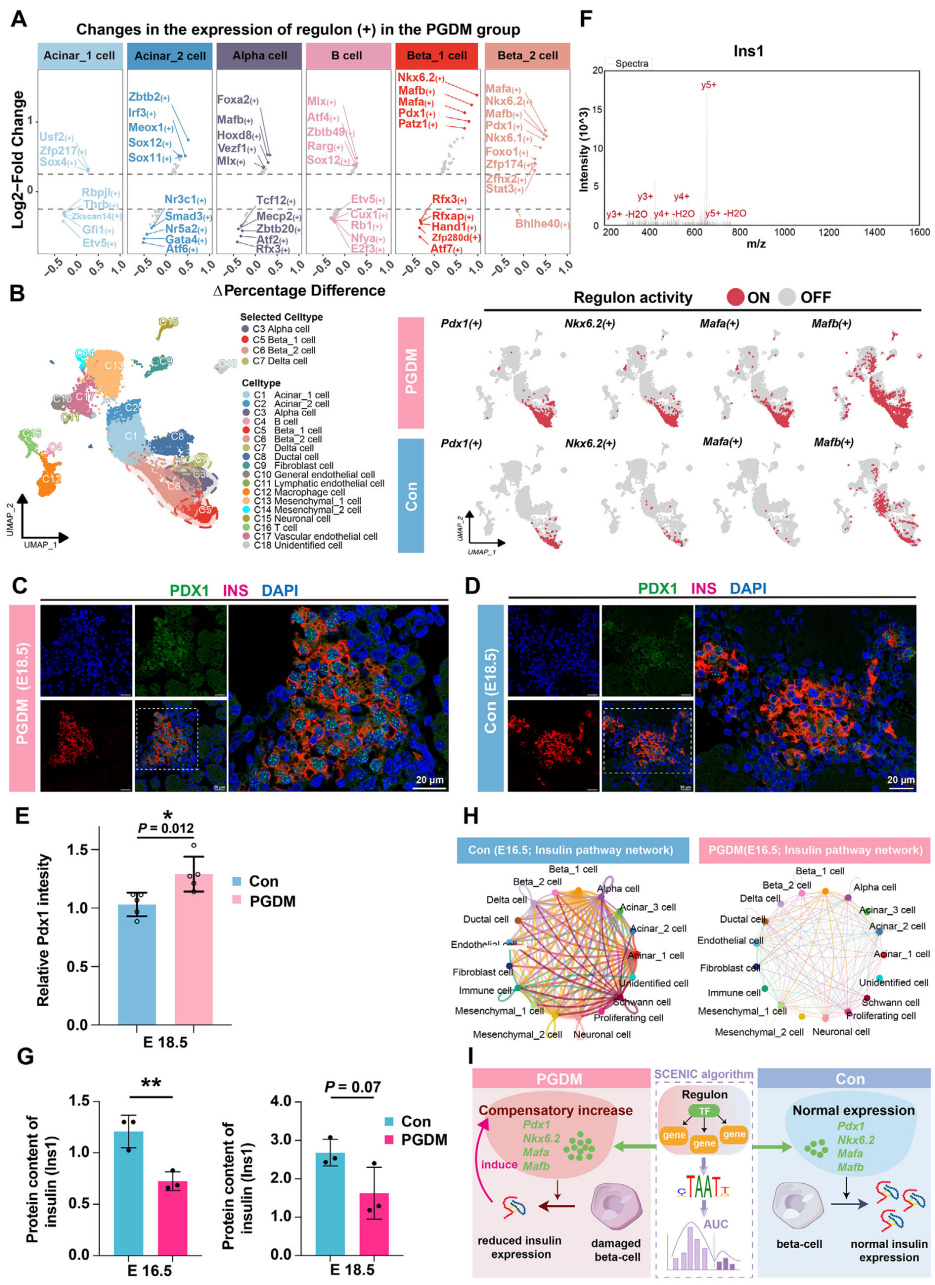
Decreased insulin secretion accompanied by the compensatory increase of Pdx1, Nkx6.2, and Mafa in the PGDM group compared with the control group. A) SCENIC analysis revealed significant changes in the expression levels of regulons in the PGDM group (E18.5) within each cluster compared with the control group (E18.5). B) UMAP plots showing the regulon activity (“on‐red,” and “off‐gray”) of *Nkx6.2(+)*, *Mafb(+)*, *Mafa(+)*, and *Pdx1(+)* in the PGDM group (E18.5) and controls (E18.5). C,D) Representative immunofluorescence images of fetal pancreas tissue sections at E18.5 for PDX1 (green), insulin (red), and nuclear stain DAPI (blue) in the PGDM group (C) and controls (D). E) Statistical analysis of immunofluorescence results. *n*  =  5/group (≈200 β‐cells/group were quantified). F) MS/MS spectrum of Ins1 using timsTOF_HT mass spectrometer G) Quantification of Ins1 proteins using quantitative proteomics data of fetal pancreas in the PGDM group (E16.5 and E18.5) and controls (E16.5 and E18.5). H) CellChat analysis showed decreased insulin signaling networks based on ligand‐receptor pairing in the PGDM group (E16.5), as compared with the control group (E16.5). I) Schematic showing regulons identified using SCENIC in the PGDM and control group. Abbreviations: SCENIC, single‐cell regulatory network inference and clustering; TF, transcription factor.

Transcription factor (TFs) played a crucial role in pancreas development and endocrine differentiation. Thus, we analyzed the activities of regulons within our snRNA‐seq data using single‐cell regulatory network interference and clustering (SCENIC) analysis. We first identified cell‐type‐specific regulons for each cluster, including *Gata4(+)*, *Nr5a2(+)*, *Etv5(+)*, *Atf6(+)*, and *Thrb(+)* in beta_1 cells, and *Jund(+)*, *Ybx1(+)*, *Mafb(+)*, *Junb(+)*, and *Cebpb(+)* in beta_2 cells (Figure S14A, Supporting Information). Next, we explored the impact of maternal PGDM on each cell population by identifying the up‐regulation of cell‐type‐specific regulons in the PGDM group compared with controls (**Figures** **3**A and S14B, Supporting Information). Notably, *Nkx6.2(+)*, *Mafb(+)*, *Mafa(+)*, and *Pdx1(+)*, which were predominantly expressed in both beta_1 and beta_2 clusters, were significantly up‐regulated in the PGDM group compared to controls (Figure 3A). More specifically, the PGDM group demonstrated higher activity of these regulons in both beta_1 and beta_2 clusters (Figure 3B).

Consistent with this observation, the PGDM group exhibited elevated gene expression levels of *Pdx1, Nkx6.1, Nkx6.2*, *Mafb*, and *Mafa* compared to the control group (Figure S14C, Supporting Information). This finding was further supported by immunofluorescence (IF) staining results, which demonstrated significantly increased expression of the PDX1 protein in the PGDM group relative to the control group (Figure 3C–E).

To further strengthen these results, we performed quantitative proteomic analysis of fetal pancreas at E16.5 and E18.5 (Figure 3F). we first carried out KEGG pathway analysis to better understand the systemic effects of maternal PGDM on the fetal pancreas. We found that the top 10 significantly up‐regulated KEGG pathways in the PGDM group (E16.5 and E18.5) included one carbon pool by folate, spliceosome, steroid hormone biosynthesis, protein digestion and absorption, hippo signaling pathway, and PI3K−Akt signaling pathway, which provided a new direction for us to further understand the effects of maternal PGDM on fetal pancreas (Figure S15, Supporting Information).

Notably, the results of proteomic analysis showed that the protein expression level of Ins1 in the PGDM group (E16.5) was significantly lower (40.0% decrease, *P* = 0.01) than that in the control group (E16.5) (Figure 3G). A decreased Ins1 expression trend (39.4% decrease, *P* = 0.07) was observed in the PGDM group (E18.5), even if statistical significance was not reached (Figure 3G). Furthermore, CellChat analysis revealed decreased insulin signaling networks based on ligand‐receptor pairing in the PGDM group (E16.5), as compared with the control group (E16.5) (Figure 3H). These results were consistent with the findings of reduced birth weight in the offspring of the PGDM group, compared to the control group (Figure S1, Supporting Information).

In conclusion, although multiple TFs including *Pdx1*, *Mafa*, and *Nkx6.2* exhibited increased activity in the PGDM group compared to the control group to compensate for insulin synthesis (Figure 3I), the impairment of endoplasmic reticulum and mitochondria (Figure 2G) caused by maternal PGDM resulted in compromised insulin secretion and beta cells.

### 2.4 The Correlation Between Maternal Metabolites and Fetal Pancreas Development in the Presence of Maternal PGDM (E18.5)

In addition, we performed a nontargeted metabolomic analysis of plasma samples from pregnant mice at E18.5, to enhance the understanding of the connection between maternal metabolism and fetal pancreatic responses. The metabolomic results showed significantly upregulated differential metabolites involved in glucose and lipid metabolism in pregnant mice with PGDM, such as Tagatose, Erythrose, D‐Allose, Fructose, Alpha‐d‐xylopyranose, D‐Glycero‐D‐gulo‐heptose, Oleoyl tyrosine, and N‐octanoylsphingosine 1‐phosphate (Figure S16, Supporting Information). Correlation analysis revealed strong correlations between maternal metabolites involved in glucose metabolism and differentially expressed proteins that regulated glucolipid metabolism in the pancreas of offspring, such as Erythrose and OSTC, D−Allose and HMCS2 (Figure S17, Supporting Information). In summary, these results offer additional mechanistic insights into the impact of maternal PGDM on fetal pancreas development.

### 2.5 Altered Intercellular Interactions Between Acinar Cells and Multiple Cell Clusters in the Presence of Maternal PGDM (E16.5 and E18.5)

**Figure 4:**
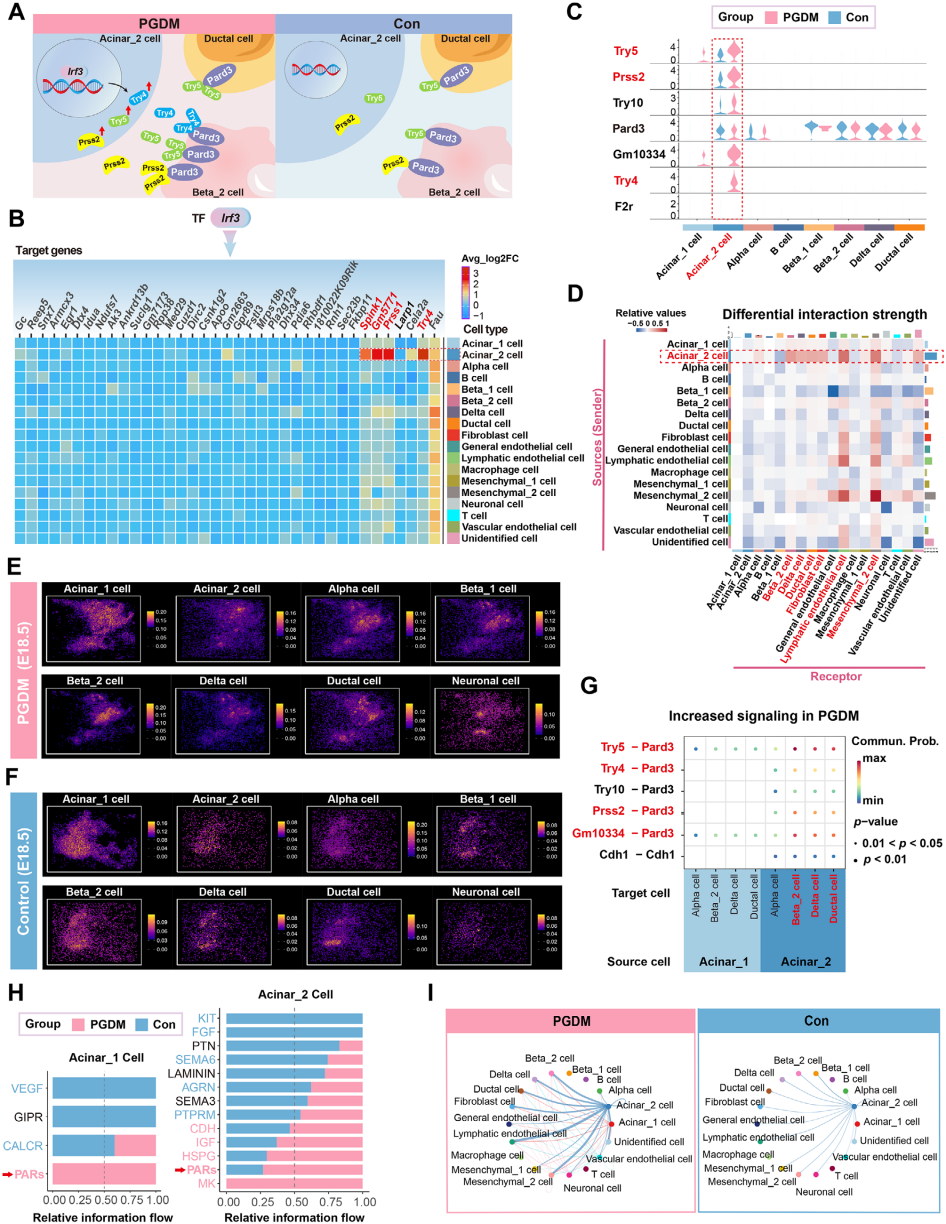
Cell–cell interaction analysis between acinar cells and multiple cell types in the presence of maternal PGDM (E18.5). A) Schematic of the intercellular communication in the PGDM and control groups. B) Heatmap showing the expression profile of candidate target genes regulated by *Irf3* in each cell population based on snRNA‐seq data (E18.5). C) Violin plots showing the expression of *Try5*, *Prss2*, *Try10*, *Pard3*, *Gm10334*, *Try4*, and *F2r* across cell populations among the PGDM (E18.5) and control (E18.5) groups, based on snRNA‐seq data (E18.5). D) Heatmap highlighting differential cell interaction strength. Red represented increased interaction strength in the PGDM group (E18.5), and blue represented decreased interaction strength in the PGDM group (E18.5), based on snRNA‐seq data (E18.5). E,F) ST data based on spots (bin 10, 10 × 10 nanoballs aggregated) showing spatial distribution of acinar_1 cells, acinar_2 cells, alpha cells, beta_1 cells, beta_2 cells, delta cells, ductal cells, and neuronal cells in the PGDM (E18.5) and control (E18.5) groups. G) CellChat analysis showed that the ligand‐receptor pairing signaling network based on snRNA‐seq data was enhanced in the PGDM group (E18.5) compared to the control group (E18.5). H) Comparison of the relative information flow of each signaling pathway between PGDM (E18.5) and control (E18.5) groups in acinar_1 and acinar_2 cells, based on snRNA‐seq data (E18.5). Significantly enriched signaling pathways in the PGDM group were colored in red text, while signaling pathways significantly enriched in the control group were colored in blue. I) Circle plots illustrating the alterations of PARs signaling pathway network among the PGDM group (E18.5) and the controls (E18.5), based on snRNA‐seq data (E18.5). Abbreviations: snRNA‐seq, single‐nucleus RNA sequencing; PARs, protease‐activated receptors.

Recent evidence has highlighted the crosstalk between acinar cells and endocrine cells (such as beta cells) in the context of diabetes.^[^ ^15^ ^]^ Based on this, we next investigated cellular interactions between acinar cells and endocrine cells during maternal PGDM (**Figure** **4**A). Initially, we observed that *Zbtb2(+)*, *Irf3(+)*, *Meox1(+)*, *Sox12(+)*, and *Sox11(+)* were significantly upregulated in the acinar_2 cell population in the PGDM group compared to controls (Figure 3A). SCENIC analysis further revealed that genes regulated by *Irf3(+)*, such as *Try4*, *Prss1*, *Spink1*, and *Gm5771*, were predominantly enriched in the acinar_2 cluster (Figure 4B). Consistent with this, we found that the PGDM group (E18.5) exhibited significantly higher expression levels of *Try4*, *Try5*, *Prss2*, and *Gm10334* within the acinar_2‐cell population compared to the control group (E18.5) (Figure 4C).

Remarkably, the CellChat analysis demonstrated that interactions between acinar_2 cells and beta_2 cells, delta cells, ductal cells, fibroblast cells, lymphatic endothelial cells, and mesenchymal_2 cells were notably enhanced in the PGDM group, with increased interaction strength compared to the control group (Figure 4D). The ST data confirmed this by showing the spatial proximity between acinar_2 cells, alpha cells, beta_1 cells, beta_2 cells, delta cells, and ductal cells (Figure 4E,F). To further investigate the mechanisms underlying the interactions of acinar_2 cells with alpha, beta_2, delta, and ductal cells, we performed ligand‐receptor analysis using CellChat at E16.5 and E18.5. The analysis revealed significant up‐regulation of signaling interactions, including *Try5‐Pard3, Try4‐Pard3, Prss2‐Pard3*, and *Gm10334‐Pard3*, in the PGDM group (E18.5) (Figure 4G). And similar results were further corroborated by snRNA‐seq data (E16.5) and ST data (E16.5) (Figures S18 and S19, Supporting Information), suggesting that these interactions might mediate communication between acinar_2 cells and other cell types under the PGDM condition (E16.5 and E18.5) (Figure 4G). For example, we conducted the CellChat analysis using snRNA‐seq data (E16.5 and E18.5) and found that Try5‐Pard3 exhibited significantly up‐regulated signaling interactions in the PGDM group compared with the control group. Remarkably, acinar_2 cell population expressed significantly higher levels of Try5 in the PGDM group compared to controls (Figure 4C), and Try5 was specifically expressed in the acinar_2 cell population. Furthermore, ST analysis revealed that Try5 and Pard3 were spatially adjacent (Figure S20, Supporting Information). Collectively, we believed the consistency of the findings strengthened our conclusions.

In addition, significant signaling pathways between the PGDM and control groups were identified based on overall information flow within inferred signaling networks, particularly the protease‐activated receptors (PARs) signaling pathway (Figure S19B, Supporting Information). Further analysis revealed that the PARs signaling pathway was highly active among acinar_1 cells and acinar_2 cells in the PGDM group (Figure 4H). Notably, the PARs signaling pathway network was significantly enhanced between the acinar_2 cluster and multiple cell clusters in the PGDM group compared to the control group (Figure 4I and Figure S18A, Supporting Information).

Overall, these findings suggested that the PARs pathway might play a critical role in mediating cell–cell interactions between acinar_2 cells and various cell clusters (such as beta_1 cells, beta_2 cells, delta cells, and ductal cells) under maternal PGDM conditions (E16.5 and E18.5).

### 2.6 Enhanced Intercellular Interactions Between Beta Cells and Endothelial Cells (ECs) in the Presence of Maternal PGDM

**Figure 5:**
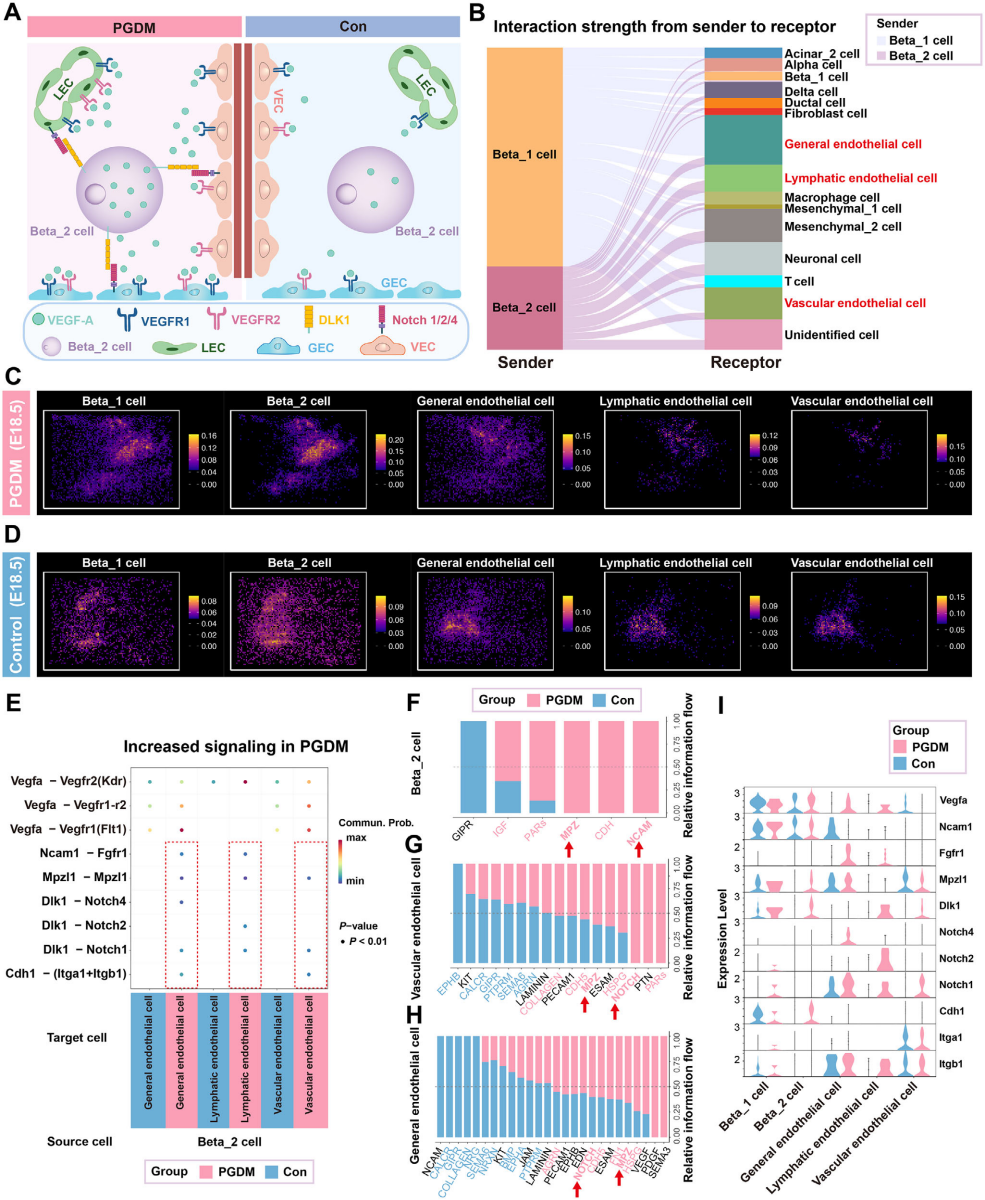
Intercellular communication networks between beta cells and endothelial cells. A) A schematic model showing the intercellular communication between beta cells and endothelial cells. B) A Sankey diagram showing the upregulated interaction strength (PGDM vs control) from beta cells to other cell types in the cell–cell communication network, based on snRNA‐seq data (E18.5). C,D) ST data based on spots (bin 10, 10 × 10 nanoballs aggregated) showing spatial distribution of beta_2 cell, GEC, LEC, and VEC in the PGDM (E18.5) and control (E18.5) groups. E) The dot plot, based on snRNA‐seq data (E18.5), displaying increased interactions between beta_2 cells, GEC, LEC, and VECs based on ligand‐receptor pairing in the PGDM group (E18.5) compared to the control group (E18.5). F‐H) Comparative relative information flow of each signaling pathway in beta_2 cells (F), VEC (G), and GEC (H) was analyzed on the basis of the snRNA‐seq data (E18.5) between PGDM and control groups. Significantly enriched signaling pathways in the PGDM group were colored in red text, and signaling pathways with blue text were significantly enriched in the control group. I) The violin plot, derived from snRNA‐seq data (E18.5), showing the gene expression levels for the indicated genes in beta cells and endothelial cells. Red represented the PGDM group; blue represented the control group. Abbreviation: GEC, general endothelial cell; LEC, lymphatic endothelial cell; VEC, vascular endothelial cell.

A previous study^[^ ^16^ ^]^ demonstrated a close physiological relationship between blood vessels and islets, with the blood vessel endothelium playing a critical instructive role in the development and differentiation of pancreatic endocrine cells. Building on this, we used snRNA‐seq data to investigate potential interactions between beta cells and ECs (**Figure** **5**A). We observed that in the PGDM group, the increased interactions between beta_2 cells and other cell types were predominantly concentrated on ECs, in contrast to the control group (Figure 5B). Spatial distribution images corroborated this by showing the proximity of beta_2 cells to general ECs, lymphatic ECs, and vascular ECs (Figure 5C,D).

To further understand the mechanisms behind these interactions, we compared ligand‐receptor interaction pairs between the PGDM and control groups, focusing on the beta_2 cells and ECs. Notably, the ligand–receptor pairs *Vegfa‐Vegfr2[kdr], Vegfa‐Vegfr1[flt1], Ncam1‐Fgfr1, Mpzl1‐Mpzl1*, and *Dlk1‐Notch (Notch1, Notch2, and Notch4*) were significantly up‐regulated in the PGDM group (Figure 5E). Information flow analysis further highlighted the activity of the MPZ, NCAM, and NOTCH pathways between beta_2 cells and ECs in the PGDM group compared to the control group (Figure 5F–H). Additionally, the PGDM group exhibited elevated expression of *Vegfa*, *Fgfr1*, *Dlk1*, *Notch1*, *Notch2*, and *Notch4* compared to the controls (Figure 5I). Moreover, ST analysis showed that Vegfa and Kdr, and Dlk and Notch2 were spatially adjacent (Figure S21, Supporting Information). These findings suggested that VEGF and Notch pathways were likely key mediators of the enhanced interactions between beta cells and ECs in the PGDM group.

### 2.7 Spatial Profiling Identified Cellular Niche Changes in Response to Maternal PGDM

**Figure 6:**
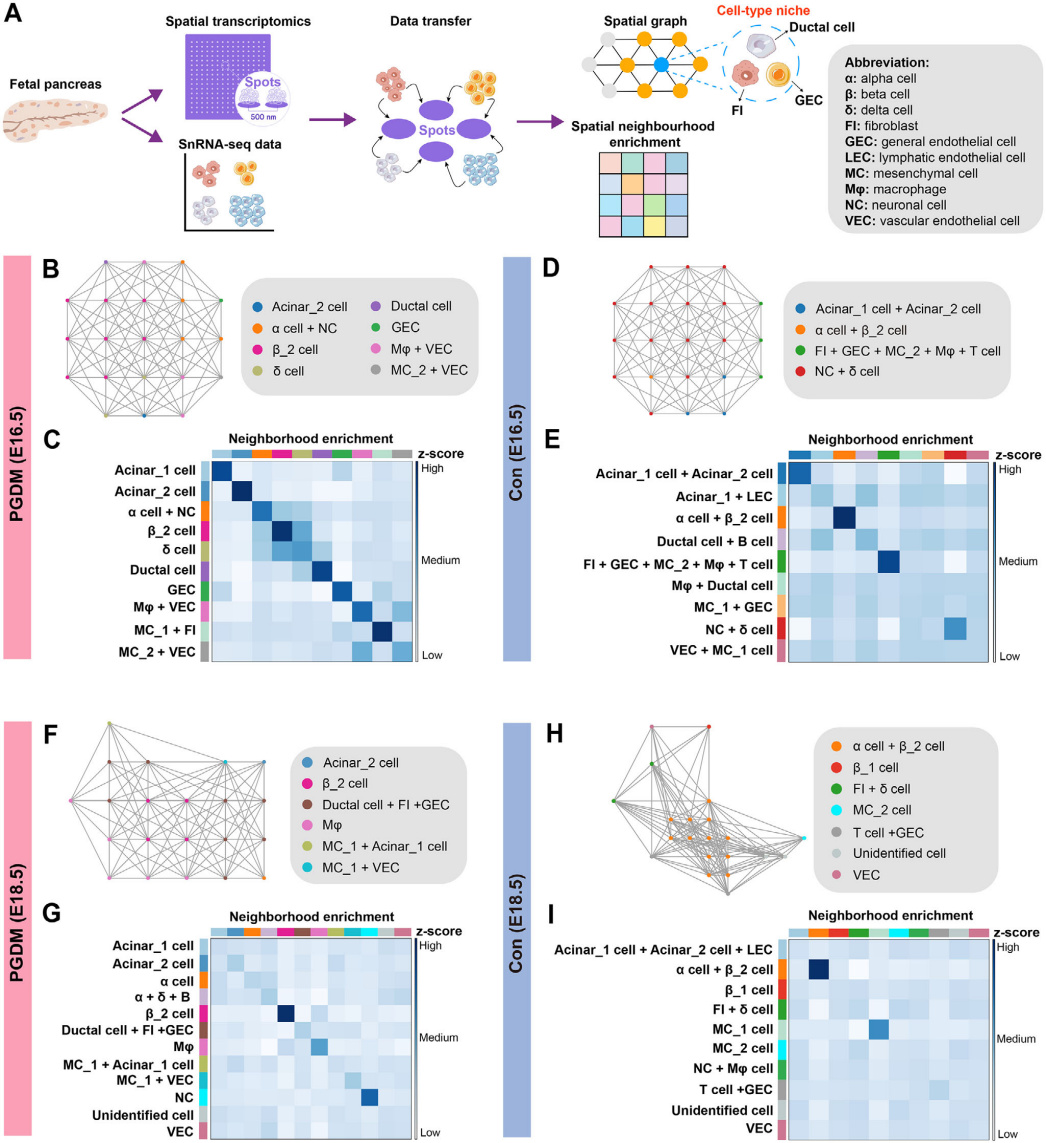
Spatial cellular niches in the fetal pancreas are affected by the maternal PGDM. A) Schematic representation of the cellular niches analysis workflow. First, cell types in stereo‐seq chips based on spots (bin 20, 20 × 20 nanoballs aggregated) were annotated with snRNA‐seq data by using “TransferData” function. Next, the spatial neighbor graph was computed to identify the cellular niches. Finally, the spatial neighborhood enrichment analysis was performed, and the enrichment score (Z‐score) was calculated. B, D, F, H) Representative spatial neighbor graph in the PGDM group (B) and control group (D) at E16.5, and the PGDM group (F) and control group (H) at E18.5. C, E, G, I) Spatial neighborhood enrichment analysis of the PGDM group (C) and control group (E) at E16.5, and the PGDM group (G) and control group (I) at E18.5.

A previous study^[^ ^17^ ^]^ found that beta cells tend to cluster in specific regions, forming insulin secretion hotspots. Additionally, gene expression levels in acinar cells, such as *Try5*, had been shown to vary based on their distance from the islets.^[^ ^15a^ ^]^ This evidence highlighted the importance of maintaining a functional cellular niche for pancreatic biology. In this context, we performed neighborhood enrichment analysis to explore the impact of maternal PGDM on local cellular niches. To improve resolution, we annotated ST data using snRNA‐seq data with anchor‐based integration method^[^ ^18^ ^]^ (**Figures** **6**A and S22, Supporting Information). Next, we constructed a spatial neighbor graph, assigning a fixed number of nearest neighbors to each spot within the ST data (Figure 6B,D,F,H). The neighborhood enrichment analysis of ST data at E16.5 revealed a tight clustering of cells within each cluster in the PGDM group (Figure 6C), compared to a more dispersed distribution observed in the control group (Figure 6E). Similar results were obtained using ST data from E18.5 (Figure 6G,I), indicating that PGDM exposure might result in altered spatial organization within the fetal pancreas. Overall, these data suggested that maternal hyperglycemia might significantly impact the local cellular niche of the fetal pancreas.

## 3 Discussion

Previous studies have demonstrated that pancreatic development is highly dependent on the interactions between islets and non‐endocrine islet cells, such as acinar cells,^[^ ^19^ ^]^ ductal cells,^[^ ^20^ ^]^ blood vessels,^[^ ^16^ ^]^ and sympathetic innervation.^[^ ^21^ ^]^ The pancreas, being a highly organized structure, is particularly vulnerable to disruptions in glucose metabolism during embryo development.^[^ ^22^ ^]^ However, little is known about the effects of maternal PGDM on the fetal pancreas, especially concerning interactions between various pancreatic cell types under maternal PGDM exposure. Utilizing snRNA‐seq data and ST analysis has enabled a more comprehensive understanding of these effects.

In our study, we successfully established a PGDM animal model to evaluate the effects of maternal PGDM on the fetal pancreas. We employed snRNA‐seq data (E16.5 and E18.5) and ST analysis (E16.5 and E18.5) to explore the underlying molecular mechanisms. We identified the cellular heterogeneity in acinar and beta cell populations based on snRNA‐seq data (E16.5 and E18.5), each displaying unique DEGs, KEGG pathways, TFs, and metabolic pathways. Additionally, maternal PGDM induced significant metabolic stress in the fetal pancreas, characterized by dilated endoplasmic reticulum cisternae and swollen mitochondria. Remarkably, our results showed that multiple TFs, including *Pdx1*, *Mafa*, and *Nkx6.2* were more active in the PGDM group than in the controls to compensate for insulin synthesis; however, maternal PGDM‐induced damage to the endoplasmic reticulum and mitochondria impaired insulin secretion and beta cells. Furthermore, the local cellular niche of the fetal pancreas and the intercellular communications between islets and non‐endocrine cells were significantly altered in the PGDM group compared to the controls.

For the first time, we utilized snRNA‐seq technology to profile the transcriptomes of individual fetal pancreatic cells in the PGDM and control groups, thus providing valuable insights into the cellular heterogeneity within the fetal pancreas under maternal PGDM conditions. We observed heterogeneity in both acinar cells and beta cells in the PGDM and control groups, underscoring the functional diversity within these cell types. The heterogeneity of acinar cells had previously been reported in both healthy individuals and adult diabetic (db/db) mice.^[^ ^11^, ^15^ ^]^ For instance, one study^[^ ^11a^ ^]^ identified three different acinar subpopulations in healthy individuals–secretory acinar cells, acinar cells with high levels of regenerating protein, and idling acinar cells. Another study^[^ ^15a^ ^]^ demonstrated the presence of two distinct acinar cells in adult db/db mice–peri‐islet acinar cells, located close to the islet, and tele‐islet acinar cells, located farther from the islet–with differing morphology and function.

Research into beta cell heterogeneity in non‐diabetic donors and adult db/db mice had also been documented.^[^ ^11^, ^23^ ^]^ A recent study^[^ ^11c^ ^]^ identified three distinct beta‐cell subtypes–β1, β2, and β3 cells–with varying biological functions in non‐diabetic individuals. Another study^[^ ^23^ ^]^ revealed that adult db/db mice (aged 6 and 10 weeks) displayed eight distinct beta cell subsets, each differing in morphology and functions. Collectively, these findings supported our results, highlighting the complexity, heterogeneity, and adaptability of acinar cells and beta cells. Further analysis was required to explore these differences.

We also noted that glycolysis/gluconeogenesis‐related pathways were significantly up‐regulated in multiple cell clusters, particularly acinar_1 cells, acinar_2 cells, beta_1 cells, and beta_2 cells, in the PGDM group (E18.5) compared to the control group (E18.5). Mitochondrial dysfunction might be responsible for the metabolic changes. Mitochondria were the principal sites for oxidative phosphorylation (OXPHOS) to produce ATP. Under maternal PGDM, mitochondrial function was impaired, causing the disruption in the OXPHOS process. Consequently, beta cells depended on glycolysis (the so‐called Warburg‐like effect) to compensate for the energy shortage for their rapid growth, proliferation, and insulin secretion.^[^ ^24^ ^]^ However, the long‐term activation of the Warburg effect produced more reactive oxygen species, leading to the dysfunction in beta cells and impairment of glucose‐stimulated insulin secretion.^[^ ^25^ ^]^

Our study also showed that maternal PGDM could disrupt the structure of endoplasmic reticulum and mitochondria compared to controls, reflecting the presence of endoplasmic reticulum stress in the fetal pancreas of PGDM group. These findings were supported by previous studies.^[^ ^26^ ^]^ For instance, one study^[^ ^26a^ ^]^ reported significantly increased DNA fragmentation in islet cells under high glucose conditions, which was mitigated by chemical inhibition of endoplasmic reticulum stress. Similarly, another study^[^ ^26c^ ^]^ using a gestational diabetes mellitus (GDM) animal model demonstrated elevated expression of 18 ribosomal genes in islets from GDM offspring, coinciding with endoplasmic reticulum stress. Interestingly, serum from exercising mice and individuals had been shown to protect beta cells from ER stress‐induced apoptosis,^[^ ^27^ ^]^ and physical exercise had been shown to restore vascular homeostasis by reducing endoplasmic reticulum stress in diabetic mice.^[^ ^28^ ^]^ While physical activity during pregnancy was an intuitive strategy for reducing endoplasmic reticulum stress in the fetal pancreas, high‐quality clinical evidence supporting its efficacy was lacking. Current Chinese guidelines recommend exercise interventions for managing GDM,^[^ ^29^ ^]^ and further studies are needed to determine whether such interventions could improve beta cell function by reducing endoplasmic reticulum stress in fetal pancreatic cells.

It had been well‐established that *Pdx1(+)*, *Nkx6.2(+)*, *Mafa(+)*, and *Mafb(+)* were critical TFs for beta cell development and maturation.^[^ ^30^ ^]^ Our data indicated that these TFs were predominantly expressed in beta cells and were significantly upregulated in the PGDM group compared to controls. As mentioned previously, distinct mitochondrial and endoplasmic reticulum damage were observed in the PGDM group. Within the endoplasmic reticulum, the native structure of insulin was formed. The close collaboration between the endoplasmic reticulum and mitochondria was essential for insulin secretion.^[^ ^31^ ^]^ Maternal PGDM‐induced damage to the endoplasmic reticulum and mitochondria impaired insulin secretion and the functions of beta cells. Therefore, insufficient insulin secretion might trigger the compensatory upregulation of Pdx1 to increase insulin production, since insulin is undeniably vital for the development of all organs and overall fetal growth of the fetus.^[^ ^9b^ ^]^ In addition, a previous study reported that the offspring of GDM mice were born with prematurely developed beta cells, largely due to the increased expression of PDX1 in the GDM group.^[^ ^22^ ^]^ Collectively, our findings suggested that maternal PGDM might result in the impairment of beta cells in offspring at birth, potentially increasing their risk of metabolic diseases, such as diabetes, in adulthood.^[^ ^32^ ^]^

Increasing evidence has shown that cell–cell communication between exocrine and endocrine cells is essential for fetal pancreatic development.^[^ ^12^, ^15^, ^33^ ^]^ We found that the PGDM group (E18.5) expressed higher levels of *Try4*, *Try5*, *Prss2*, and *Gm10334* within the acinar_2‐cell population compared to the controls (E18.5), which might relate to islet expansion.^[^ ^15a^ ^]^ Previous studies^[^ ^15a^ ^]^ also demonstrated significantly higher mRNA levels of *Try5* in “peri‐islet” acinar cells, located near the islet, compared to “tele‐islet” acinar cells, which were further from the islet, in adult db/db mice. This zonated gene expression signature was not observed in age‐matched control mice. Furthermore, the same study^[^ ^15a^ ^]^ reported significant increases in the expression levels of *Try4* (4.4‐fold), *Try5* (8.5‐fold), *Try10* (9.2‐fold), *Prss1* (1162‐fold), *Prss2* (1.07‐fold), and *Prss3* (338‐fold) in db/db mice compared to controls. These findings supported our observations.

By analyzing ligand‐receptor pair communication, we also found a significant increase in interactions between acinar_2 cells and beta_2 cells via the *Try5‐Pard3* and *Prss2‐Pard3* signaling pathways in the PGDM group (E16.5 and E18.5) compared to the control group (E16.5 and E18.5). Additionally, we observed enrichment of the PARs pathway in the PGDM group (E16.5 and E18.5) but not in controls (E16.5 and E18.5). It had been shown that PARs (PAR1‐4) were important mediators of acinar‐beta‐cell crosstalk and played a role in maintaining beta‐cell homeostasis.^[^ ^15b^ ^]^ These findings extended prior research, indicating that the *Try5‐Pard3, Try4‐Pard3*, and *Prss2‐Pard3* signaling pathways were critical to acinar‐beta‐cell crosstalk. Further elucidation of these crosstalk mechanisms was crucial for understanding alterations in fetal pancreatic cells in response to maternal PGDM.

Though ECs were known to provide essential developmental signals supporting beta‐cell development,^[^ ^16^ ^]^ the mechanisms underlying beta cell‐EC interactions in fetuses exposed to maternal PGDM remained poorly understood. In our study, we discovered upregulated expression of ligand–receptor pairs between beta_2 cells and ECs in the PGDM group (E18.5) compared to controls (E18.5), particularly, the *VEGFA‐VEGFR* pair. Tosti et al.^[^ ^11a^ ^]^ reported that ECs were located closest to pancreatic islets among pancreatic non‐epithelial cells in healthy individuals, suggesting that ECs played a critical role in beta‐cell proliferation and regeneration in a VEGFA‐dependent manner.^[^ ^34^ ^]^ After VEGFA induction, ECs proliferated in mouse islets, recruited circulating monocytes that differentiated into macrophages,^[^ ^34^ ^]^ and activated various growth factors and cytokines that promoted beta‐cell regeneration.^[^ ^34^ ^]^ These findings suggested that further research was needed to determine the role of the *VEGFA‐VEGFR* signaling pathway in beta cell‐EC interactions in maternal PGDM.

Maintaining niche homeostasis was essential for pancreatic development, yet the impact of maternal PGDM on cellular niches remained unidentified. One study^[^ ^11a^ ^]^ quantified islet distribution during pancreas morphogenesis in healthy human tissue, reporting that the minimum distance between islet centroids is 400 µm. Another study^[^ ^35^ ^]^ used a multi‐spectral imaging mass cytometry approach to analyze cellular niches in non‐diabetic (ND, *n* = 13) and type 2 diabetes mellitus (T2D, *n* = 16) populations. In T2D pancreases, the study^[^ ^35^ ^]^ found that certain cell types, including alpha cells, beta cells, delta cells, ductal cells, macrophages, and CD8 T cells, exhibited closer spatial proximity than in ND pancreases. Our neighborhood enrichment analysis of ST data showed tight distribution within each cluster in the PGDM group, though further investigation was needed to determine the significance of these findings.

There were several limitations to this study. First, although we integrated multi‐omics data to analyze the effects of intrauterine hyperglycemia on the fetal pancreas during critical developmental periods (E16.5 and E18.5), we did not analyze the pancreas at earlier stages (E8.5‐14.5) due to the small dimensions of the pancreas at these stages, which made multi‐omics analysis challenging. For example, even at E18.5, the pancreas was only 5 mm in length (Figure 1A). Second, pancreatic exocrine tissues contained high levels of hydrolytic enzyme activities,^[^ ^11a^ ^]^ making the samples used for multi‐omics analysis demanding. The relatively small number of samples necessitated further validation of our findings. We utilized IF and TEM to independently confirm the multi‐omics data. Third, the impact of maternal PGDM on ribosomal genes needed to be interpreted with caution, as these ribosomal genes were not ideal markers for distinguishing cellular differences. Further studies were required to validate our findings at the single‐cell level, such as single‐cell proteomics. Additionally, we induced severe hyperglycemia using STZ intraperitoneal injection^[^ ^2b^ ^]^ because severe hyperglycemia posed significant risks to the fetus. However, the effects of varying degrees of pregestational hyperglycemia (e.g., moderate vs severe) on pancreatic cells remained unclear.^[^ ^36^ ^]^ Further investigation was warranted. Finally, ethical and practical constraints limited clinical trials in pregnant women, making animal models the primary strategy for studying fetal pancreas development during pregnancy. Future studies with more human fetal pancreas samples were needed to authenticate our findings and expand our understanding of fetal pancreas development in maternal PGDM.

## 4 Conclusion

Taken together, for the first time, we conducted a comprehensive investigation of the effects of intrauterine hyperglycemia on fetal pancreas using integrated snRNA‐seq and ST approaches. We observed pronounced cellular responses to metabolic stress, significantly decreased insulin secretion accompanied by the compensatory increase of Pdx1, Nkx6.2 and Mafa in PGDM offspring at birth, altered cell‐cell communications among multiple cell types, and distinct changes in cellular niches in the PGDM group compared to controls. These findings provide a foundation for further exploration of intrauterine hyperglycemia's effects on fetal pancreas and have implications for future PGDM‐related research and treatment strategies aimed at mitigating fetal pancreas injury during pregnancy.

## 5 Experimental Section

### PGDM Mouse Model

C57BL/6J mice were purchased from Beijing Weitong Lihua Experimental Animal Technology Co. Ltd. (Beijing, China). All mice were acclimatized to laboratory conditions (22–26 °C with a 12‐h light/dark cycle) for one week before the experiment. The Animal Ethics Committee of Peking University First Hospital approved all animal procedures (Approval No. J2023052). For the experimental group (PGDM group, Figure 1A), 8–9–week–old female mice were administered an intraperitoneal injection of streptozotocin (STZ, # S0130, Sigma–Aldrich) at a dose of 80 mg/kg body weight/day for three consecutive days, as previously described.^[^ ^37^ ^]^ The STZ was freshly dissolved in sodium citrate buffer (0.1 mol L^−1^, pH 4.5, Solarbio). Control group mice (Figure 1A), also 8–9–weeks–old, were injected intraperitoneally with sodium citrate buffer (0.1 mol L^−1^, pH 4.5, sterile, Solarbio). One week after the final injection, random glucose levels were measured using an Accu‐chek Performa glucometer (Roche, Germany). The PGDM model was confirmed when two consecutive random blood glucose readings exceeded 14 mmol L^−1^. After confirming successful PGDM induction, female mice were housed with male mice for overnight mating (female: male  =  2:1). The presence of a vaginal plug the following morning was considered embryonic day (E) 0.5. Pregnant mice were sacrificed on embryonic days E12.5, E14.5, E16.5, and E18.5 (Figure S1, Supporting Information), and fetal pancreas tissue was rapidly dissected using a stereomicroscope (Leica).

### Sample Preparation of snRNA‐seq

Fetal pancreas samples were collected from PGDM and control groups (Figure S2, Supporting Information). Nuclei were isolated from snap‐frozen tissue according to the manufacturer's protocols. Briefly, the frozen fetal pancreas was homogenized in homogenization buffer. After homogenization, filtration, and concentration, nuclei were separated and collected.

### snRNA‐seq Library Preparation and Sequencing

snRNA‐seq libraries were prepared using the Kit v3.1 (#1 000 268; 10× Genomics) following the manufacturer's instructions. Briefly, frozen fetal pancreas tissues were ground using a tissue grinder, and the nuclei were extracted using lysis buffer. The nuclei suspension was subjected to filtration (30 µm), washing, and density gradient centrifugation. The purified nuclei were then mixed with a reverse‐transcription reagent mix (#1 000 123; 10× Genomics) and loaded onto a microfluidic chip for library construction. Libraries were sequenced on a NovaSeq platform (Illumina). Data quality metrics were summarized in Table S3 (Supporting Information).

### snRNA‐seq Data Processing

Raw sequencing data were aligned to the mouse genome (mm10) and processed using Cell Ranger (version 7.1.0). The resulting expression matrix was analyzed using the Seurat package (version 4.4.0) in R (version 4.3.2). Low‐quality nuclei were filtered out based on the following criteria: nFeature_RNA > 200, nFeature_RNA < 4000, percent.mt < 20, and nCount_RNA < 30 000.^[^ ^38^ ^]^ The filtered data were normalized using the “SCTransform” function in Seurat. Batch effects between samples were corrected using Harmony (version 1.2.0) with default parameters in Seurat “RunHarmony” function. Clustering was then performed using the “FindClusters” function with a resolution parameter set to 0.5. Dimensionality reduction was carried out using the “RunUMAP” function with dimensions 1:20. The final snRNA‐seq library contained 21657 nuclei (E18.5) and 38237 nuclei (E16.5), representing all major fetal pancreatic cell types.

### Identification of DEGs

DEGs analysis between the PGDM and control groups was performed within each single‐nucleus cluster using the “FindMarkers” function in Seurat, based on the Wilcoxon rank‐sum test. DEGs were defined as genes with a | log2 fold change (FC)| > 0.25 and an adjusted *P*‐value < 0.05.

### GO Enrichment Analysis

Differential expression analysis between PGDM and control groups was carried out with the R Seurat package using the “FindMarkers” function. For each cell cluster, the top 50 upregulated genes (avg_log2FC > 1 and *P*_val_adj < 0.05) were filtered as the selected DEGs between the PGDM and control groups. Subsequent GO enrichment analysis between PGDM and control groups based on the selected DEGs was conducted using the clusterProfiler package (version 4.6.2).^[^ ^39^ ^]^

### KEGG Pathway Enrichment Analysis

KEGG pathway enrichment analysis between PGDM and control groups within each cluster was conducted using the clusterProfiler package (version 4.6.2).^[^ ^39^ ^]^ KEGG terms with Benjamini‐Hochberg adjusted *P*‐values < 0.05 were considered significantly enriched.

### Spatial Transcriptomics

Reagents were obtained from the STOmics Gene Expression kit (#101KT114, BGI, Shenzhen, China), and the procedure was performed according to the manufacturer's instructions.

### Sample Preparation and Tissue Permeabilization

Fetal pancreas tissues were embedded in OCT and flash‐frozen in liquid nitrogen. Cryosections (10 µm thick) were then mounted onto the surface of Stereo‐seq chips. The chips were incubated at 37 °C for 5 min and then fixed in cold methanol (−20 °C) for 30 min. Subsequently, permeabilization was performed using PR enzyme (#F1‐230828, BGI, Shenzhen, China).

### cDNA Library Preparation for Sequencing

Following tissue permeabilization, reverse transcription mix (#101KT114, BGI, Shenzhen, China) was added to synthesize cDNA. After reverse transcription, tissues were removed, and cDNA release mix (#101KT114, BGI, Shenzhen, China) was applied to release the cDNA from the chip surface. The supernatant containing cDNA was purified using DNA clean beads (#N411‐02, Vazyme, Nanjing, China) and transferred into 1.5 mL EP tubes for polymerase chain reaction (PCR) amplification.

The amplified PCR products were then fragmented using Fragmentation Mix (#101KL114, BGI, Shenzhen, China), and spatial libraries were constructed with the library kit (#101KL114, BGI, Shenzhen, China). Finally, the libraries were sequenced on the BGI‐T7 sequencing platform (MGI, Shenzhen, China). Data quality for ST data was shown in Table S4 (Supporting Information).

### ST Data Processing

Raw FASTQ files were processed using the SAW software (https://github.com/STOmics/SAW) and converted into gene expression matrices for further analysis. ST data were analyzed using the Seurat package (version 4.4.0) in R (version 4.3.2). Briefly, the count matrices were normalized and scaled using the “SCTransform” method. Dimensionality reduction was then performed via the “RunPCA” function. Clustering and visualizations of bins were conducted with the “FindNeighbours”, “FindClusters”, and “RunUMAP” functions. Relative gene expression features were visualized using the “SpatialFeaturePlot” function in Seurat (version 4.4.0).

Stereo‐seq chips had a spot diameter of ≈220 nm, with a subcellular resolution of 500 nm between spots.^[^ ^40^ ^]^ The bin sizes were as follows: bin 10 (10 × 10 nanoballs were aggregated) measured 5 µm × 5 µm, bin 20 (20 × 20 nanoballs were aggregated) measured 10 µm × 10 µm, and bin 50 (50 × 50 nanoballs were aggregated) measured 25 µm × 25 µm.^[^ ^41^ ^]^ Bins 10, 20, and 50 were selected based on the specific research objectives.^[^ ^40^ ^]^ The specific bin size used was reported in the corresponding figure legend.

### The Visualization of the Spatial Distribution for Specific Cell Type

To visualize the spatial distribution of specific cells of the ST data (E18.5), spots were annotated based on snRNA‐seq data in Seurat. And then the first top 150 markers in each cell type from snRNA‐seq data were calculated by the “FindAllMarkers” function as cell‐type‐specific signature genes. Finally, the cell type signature score was calculated for spots (bin 10) by using UCell package and visualized.^[^ ^42^ ^]^

### Cell‐Cell Communication Analysis

Cell‐cell communication networks were analyzed through ligand–receptor interactions using the CellChat R package (version 1.6.1) based on snRNA‐seq data.^[^ ^43^ ^]^ First, the “rankNet” function in CellChat was used to compare information flow between the PGDM and control groups across major clusters. Next, significant intercellular communications between cell populations were predicted for each signaling pathway using the “computeCommunProbPathway” function. Dominant senders and receivers in the intercellular communication network were then identified.

### Investigation of Transcription Factor Activity

To identify differentially regulated regulons (i.e., TF and their putative target genes) between PGDM and control groups within each cluster, the pySCENIC analysis pipeline (version 0.12.1) was used.^[^ ^44^ ^]^ The input matrix for pySCENIC consisted of gene expression counts for all cells. Briefly, gene co‐expression network analysis, TF‐motif enrichment, and regulon activity were performed using the “pyscenic grn”, “pyscenic ctx”, and “pyscenic aucell” functions, respectively. The Wilcoxon rank‐sum test was used to identify specific regulons that were differentially active between PGDM and control groups within each cluster. Binarized regulon activity was then projected onto UMAP trajectories.

### Evaluation of Metabolic Heterogeneity at Single‐Nucleus Resolution

Single‐nucleus metabolic activity was quantified using the scMetabolism (version 0.2.1) R package.^[^ ^45^ ^]^ The metabolic gene set signature scores, based on KEGG pathways, were evaluated using the “AUCell” method. These signature scores were then imported into the Seurat S4 object for visualization.

### PAGA Trajectory Analysis

PAGA^[^ ^46^ ^]^ was applied to assess the global connectivity topology among the selected cell clusters. Two distinct snRNA‐seq data (E16.5 and E18.5) were first integrated and corrected the batch effects with “sce.pp.harmony_integrate” function.^[^ ^47^ ^]^ The “scanpy.tl.paga” function was applied then to construct the PAGA graph with default settings.

### Pseudo‐Time Analysis

The Monocle2 (version 2.28.0) R package^[^ ^48^ ^]^ was applied to analyze pseudo‐time trajectories to predict developmental processes of acinar cells, beta cells and ductal cells. Briefly, the input to Monocle2 was created from the UMI count matrix of the highly variable genes using the “newCellDataSet” function. The “DDRTree” method was applied in the “reduceDimension” function to reduce dimensions, and “plot_cell_trajectory” function was performed for visualization.

### Cell‐Type Niche Analysis

A spatial graph was computed for each sample using the spatial coordinates of bin 20 to identify neighboring spots. Two spots were connected by an edge if they were adjacent in the spatial map. The spatial neighbor graphs were generated using the “sq.gr.spatial_neighbors” function, and neighborhood enrichment was analyzed using the “sq.gr.nhood_enrichment” function.^[^ ^49^ ^]^

### TEM

TEM was performed on fetal pancreas samples to examine ultrastructural details. The samples were fixed in 2.5% glutaraldehyde, dehydrated in ethanol, embedded in resin, sectioned, and counterstained. The ultrastructure of the pancreas tissues was observed using a TEM (TECNAI G2 20 TWIN, FEI, Czech Republic) at magnifications of 4400x, 10000x, and 25500x.

### Quantitative Proteomics

To assess the robustness of the SnRNA‐seq data, quantitative proteomic analysis of fetal pancreas samples (E16.5 and E18.5) was performed. Briefly, the main analysis steps were described below. The protein was extracted from fetal pancreas (E16.5 and E18.5), and DIA quantitative proteomics analysis was performed by Beijing Qinglian Biotech Co.,Ltd (Beijing, China). MS spectra were acquired with mass range 300–1500 m z^−1^ with a resolution of 60000 FWHM (1222 m z^−1^). Peptides were measured by a timsTOF_HT mass spectrometer (Bruker, Bremen, Germany). Then, data were processed by database searching against uniprot Mus musculus UP000000589 proteome database (54822 target sequences downloaded on 2024‐03‐07) using Spectronaut software (Biognosys, version 19.0). Lastly, based on the differentially expressed proteins (fold change cutoff = 1.2 and *P*‐value < 0.05) between PGDM and control (E16.5 and E18.5) groups, the biological functions of differentially expressed proteins were evaluated by using KEGG pathway analysis.

### Metabolomics Analysis

Plasma samples from 12 pregnant mice at E18.5 were prepared, and 180 µL of supernatant was transferred for LC‐MS analysis after centrifugation. The untargeted metabolomics analysis was performed by Metware Biotechnology Co., Ltd (Wuhan, China). The MS analysis was operated in the positive ion mode and negative ion mode using full scan analysis over m/z 75–1000 at 35 000 resolution. Differential metabolites between PGDM and control (E18.5) groups were identified by VIP score (VIP > 1) and *P*‐value (*P*‐value < 0.05, Student's *t* test).

### Tissue Immunofluorescence (IF)

Fetal pancreas samples (4 µm thick) were baked for 60 min at 60 °C, then dewaxed and rehydrated. Antigen retrieval was performed in sodium citrate buffer (pH  =  6.0, #ZLI‐9064, Zhongshan Golden Bridge, Beijing, China) under high pressure. The sections were then incubated in endogenous peroxidase blocking solution (#P0100B, Beyotime, Shanghai, China) for 15 min at room temperature, followed by blocking with 5% BSA for 30 min at room temperature. The samples were incubated overnight at 4 °C with primary antibodies against Pdx1 (#5679, RRID: AB_10 706 174, 1:200 dilution, Cell Signaling Technology, USA) and insulin (#GB12335, RRID: AB_2 923 360, 1: 1500 dilution, Servicebio, Wuhan, China). The next day, sections were incubated with 488‐labeled goat anti‐rabbit IgG (#A0423, RRID: AB_2 891 323, 1: 600 dilution, Beyotime, Shanghai, China) and Cy3‐labeled goat anti‐mouse IgG (#A0521, RRID: AB_2 923 334, 1: 600 dilution, Beyotime, Shanghai, China). Finally, sections were stained with 4,6‐diamidino‐2‐phenylindole (DAPI, #C1002, 1: 1000 dilution, Beyotime, Shanghai, China) for 5 min at room temperature, and immunofluorescent images were captured using a fluorescence microscope (DMi 8; Leica, Microsystems, Germany).

### Statistical analysis

Data were presented as mean ± SD. Statistical significance was assessed using the Wilcoxon rank‐sum test for nonparametric data and the *t* test for parametric data. A *P* value of less than 0.05 was considered statistically significant. All statistical analysis were conducted using R (version 4.3.2), unless otherwise specified.

## Conflict of Interest

The authors declare no conflict of interest.

## Author Contributions

Y.D. and S.W. contributed equally to this work. Y.D. and H.Y. conceived and designed the research. Y.D. and S.W. performed the experiments. Y.D. and Z.Y. performed the analysis of snRNA‐seq and spatial transcriptomics data. Y.D. and H.Y. wrote the manuscript. All authors helped revise the manuscript.

## Supporting information



Supporting Information

## Data Availability

The data that support the findings of this study are openly available in China National GeneBank (CNGB) database at http://db.cngb.org/cnsa/project/CNP0006169_bbec3044/reviewlink/, the accession numbers are CSE0000432 and CSE0000433.
