# Supplementary material for: Integrating Spatial and Single‐Nucleus Transcriptomic Data to Assess the Effects of Intrauterine Hyperglycemia on Fetal Pancreatic Development
Source: Adv Sci (Weinh). 2025 May 19;12(22):2411126. doi: 10.1002/advs.202411126 (PMC12165054; doi:10.1002/advs.202411126)
Supplement: Supplementary file 1 — Supporting Information [file ADVS-12-2411126-s001.pdf]

ADVANCED SCIENCE
Open Access

Supporting Information

for *Adv. Sci.*, DOI 10.1002/advs.202411126

Integrating Spatial and Single-Nucleus Transcriptomic Data to Assess the Effects of Intrauterine Hyperglycemia on Fetal Pancreatic Development

*Yu Deng, Shuting Wan, Zan Yuan and Huixia Yang**

Supporting Information

Integrating spatial and single-nucleus transcriptomic data to assess the effects of intrauterine hyperglycemia on fetal pancreatic development

*Yu Deng, Shuting Wan, Zan Yuan, Huixia Yang**

This PDF file includes:

Figures S1 to S22

Tables S1 to S4

References (1 – 56)

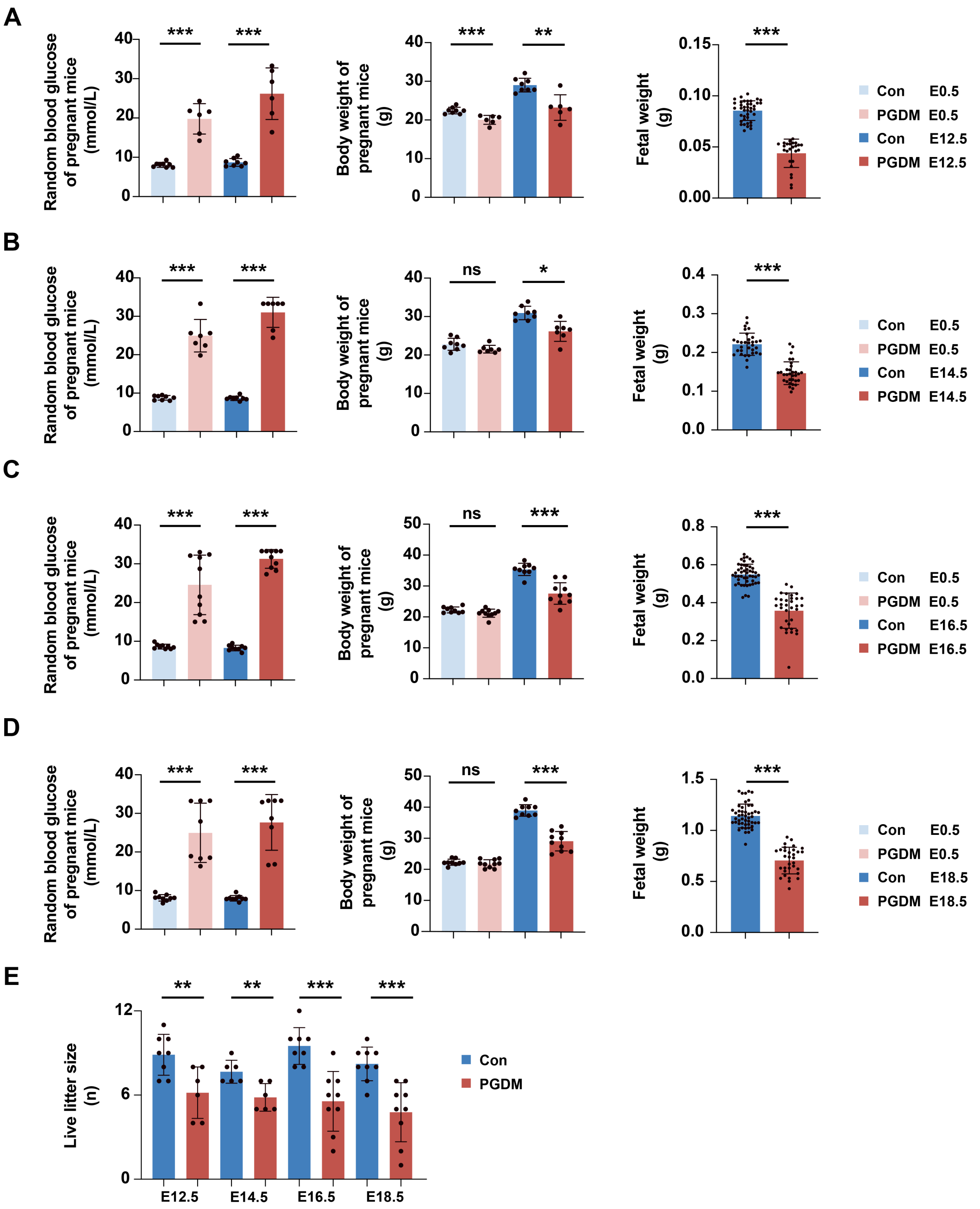

Figure S1. Maternal and offspring characteristics of the PGDM and control (Con) group at E12.5, E14.5, E16.5 and E18.5, related to Figure 1.

(A-E) Random blood glucose levels of pregnant mice, body weights of pregnant mice and fetal mice, and live fetuses per litter at different developmental time points (E0.5, E12.5, E14.5, E16.5 and E18.5) between the PGDM and Con group. Data represented as the mean ± SD. *P* values were calculated by t-tests. * $P < 0.05$, ** $P < 0.01$, *** $P < 0.001$ versus the Con group. Abbreviation: E0.5, embryonic day E0.5; E12.5, embryonic day E12.5; E14.5, embryonic day E14.5; E16.5, embryonic day E16.5; E18.5, embryonic day E18.5; ns, not significant.

A E16.5

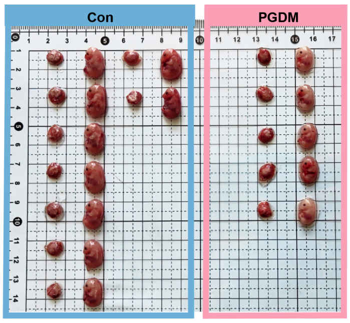

B E16.5

Cell_type	PGDM Sample (No.1-3)	Con Sample (No.4-5)	Con Sample (No.6-7)	
Acinar_1 cell	4262	1260	1322	6844
Acinar_2 cell	1621	1505	2425	5551
Acinar_3 cell	692	1676	1740	4108
Mesenchymal_1 cell	1318	958	1214	3490
Ductal cell	1208	784	1184	3176
Proliferating cell	1335	489	1277	3101
Mesenchymal_2 cell	719	1041	889	2649
Endothelial cell	412	865	806	2083
Beta_1 cell	515	742	724	1981
Alpha cell	625	632	506	1763
Immune cell	159	526	383	1068
Beta_2 cell	342	257	267	866
Fibroblast cell	96	256	231	583
Schwann cell	82	178	102	362
Neuronal cell	65	214	50	329
Delta cell	33	56	57	146
Unidentified cell	5	70	62	137
	13489	11509	13239	38237

C E18.5

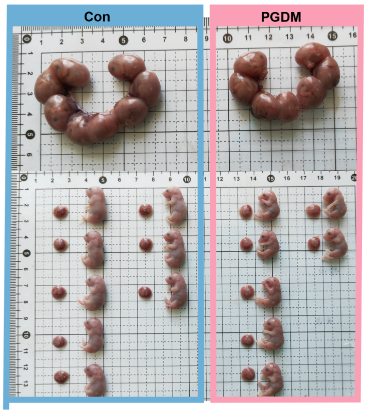

D E18.5

Cell type	PGDM Sample (No.8-11)		Con Sample (No.12-13)	Con Sample (No.14-15)	
Acinar_1 cell	973	1277	1712	2776	6738
Acinar_2 cell	125	171	316	767	1379
Alpha cell	261	326	204	297	1088
B cell	9	34	30	20	93
Beta_1 cell	2	8	317	452	779
Beta_2 cell	627	1168	166	292	2253
Delta cell	75	76	91	104	346
Ductal cell	232	402	406	617	1657
Fibroblast cell	126	195	123	133	577
General endothelial cell	70	89	60	68	287
Lymphatic endothelial cell	21	34	26	35	116
Macrophage cell	226	288	236	309	1059
Mesenchymal_1 cell	548	819	513	1015	2895
Mesenchymal_2 cell	11	7	38	67	123
Neuronal cell	71	89	81	80	321
T cell	43	168	181	133	525
Vascular endothelial cell	210	323	211	418	1162
Unidentified cell	44	49	54	112	259
	3674	5523	4765	7695	21657

E

	PGDM	Con
SnRNA_E16.5	sample(No.1-3)	sample(No.4-7)
SnRNA_E18.5	sample(No.8-11)	sample(No.12-15)
ST_E16.5	sample(No.16)	sample(No.17)
ST_E18.5	sample(No.18-19)	sample(No.20-21)

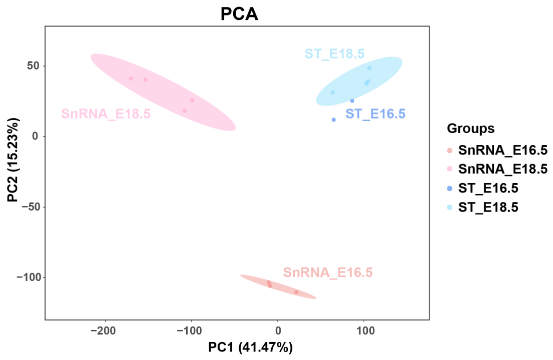

Figure S2. The sample collection of single nuclei from the fetal pancreas (E16.5 and E18.5), related to Figure 1.

(A) Representative images of E16.5 fetuses and placentas from the PGDM and control (Con) group. Pancreatic tissues were stored in RNAlater, showing some degree of dehydration. **(B)** Distribution of 38237 single nucleus in each cluster from the fetal pancreas (E16.5). **(C)** Representative images of E18.5 conceptuses (upper panel), fetuses (lower panel), and placentas (lower panel) from the PGDM and control (Con) group. **(D)** Distribution of 21657 single nucleus in each cluster from the fetal pancreas (E18.5). **(E)** PCA of all samples used in this study. Twenty-one samples included in analysis were summarized in left panel. Abbreviation: PCA, principal component analysis.

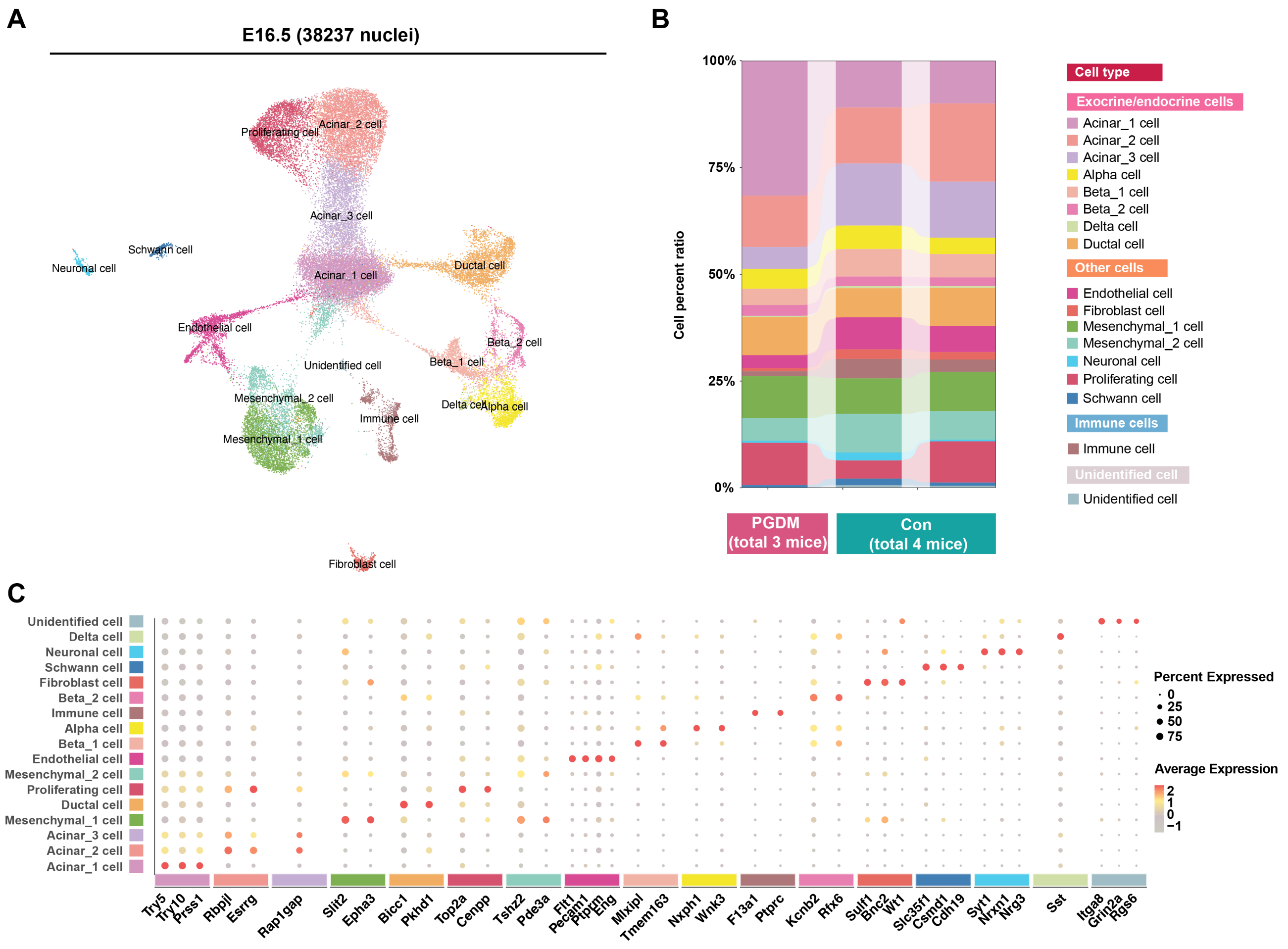

Figure S3. Global single-nucleus transcriptome map of fetal pancreas (E16.5).

(A) Uniform manifold approximation and projection (UMAP) representation of the fetal pancreas (E16.5). **(B)** Bar graph showing the cluster composition of the PGDM and control (Con) groups at E16.5 in this study. **(C)** Dotplot showing the expression levels of specific marker genes in each cell cluster at E16.5.

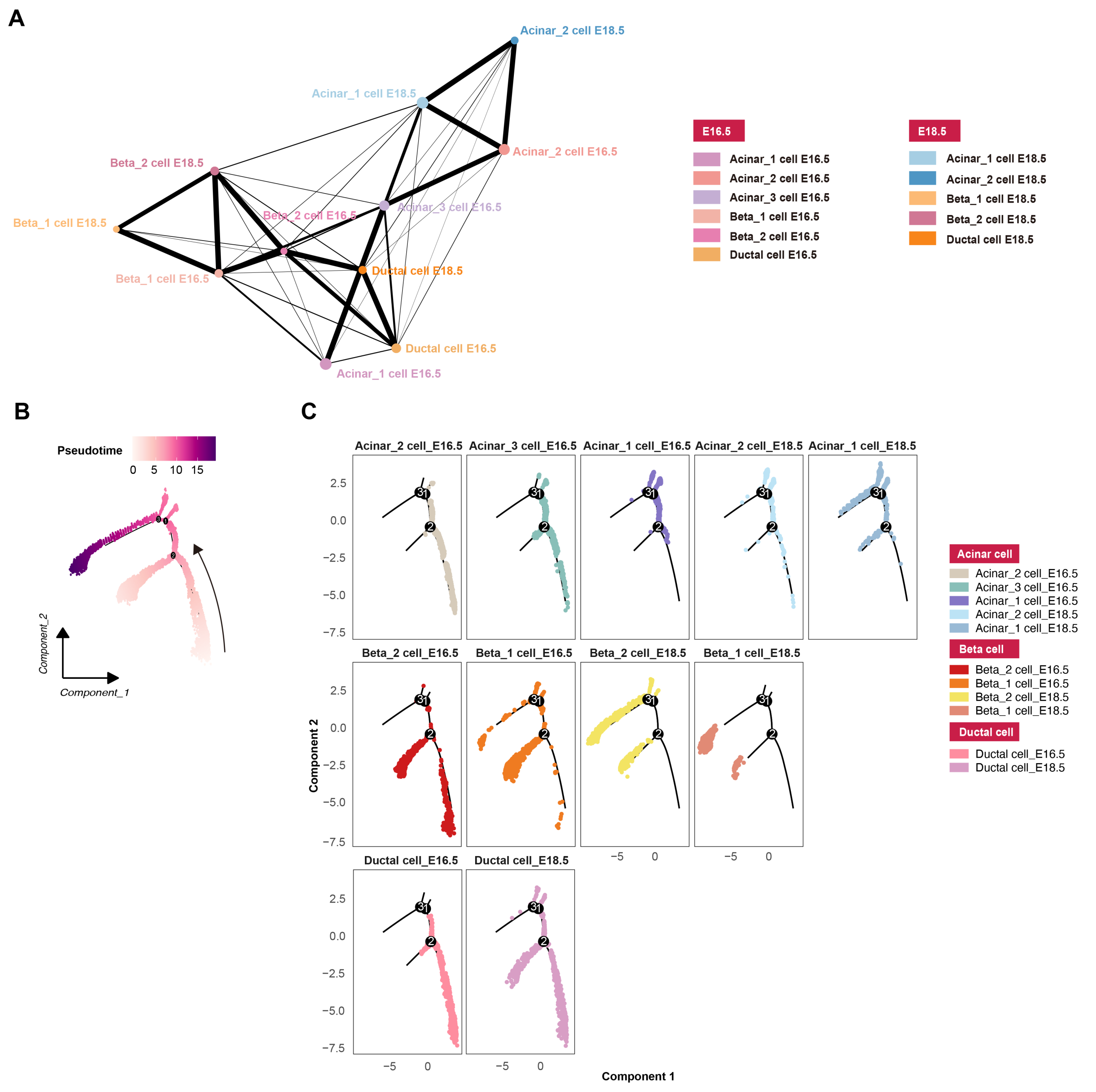

Figure S4. The PAGA and pseudo-time trajectory analysis among acinar and beta subpopulations.

(A) PAGA trajectory analysis among acinar and beta subpopulations. Each node represented a cell partition (cluster) and thicker edges indicated stronger connectedness between clusters. **(B-C)** Pseudo-time trajectory analysis among acinar and beta subpopulations using Monocle2. Abbreviations: PAGA, partition-based graph abstraction.

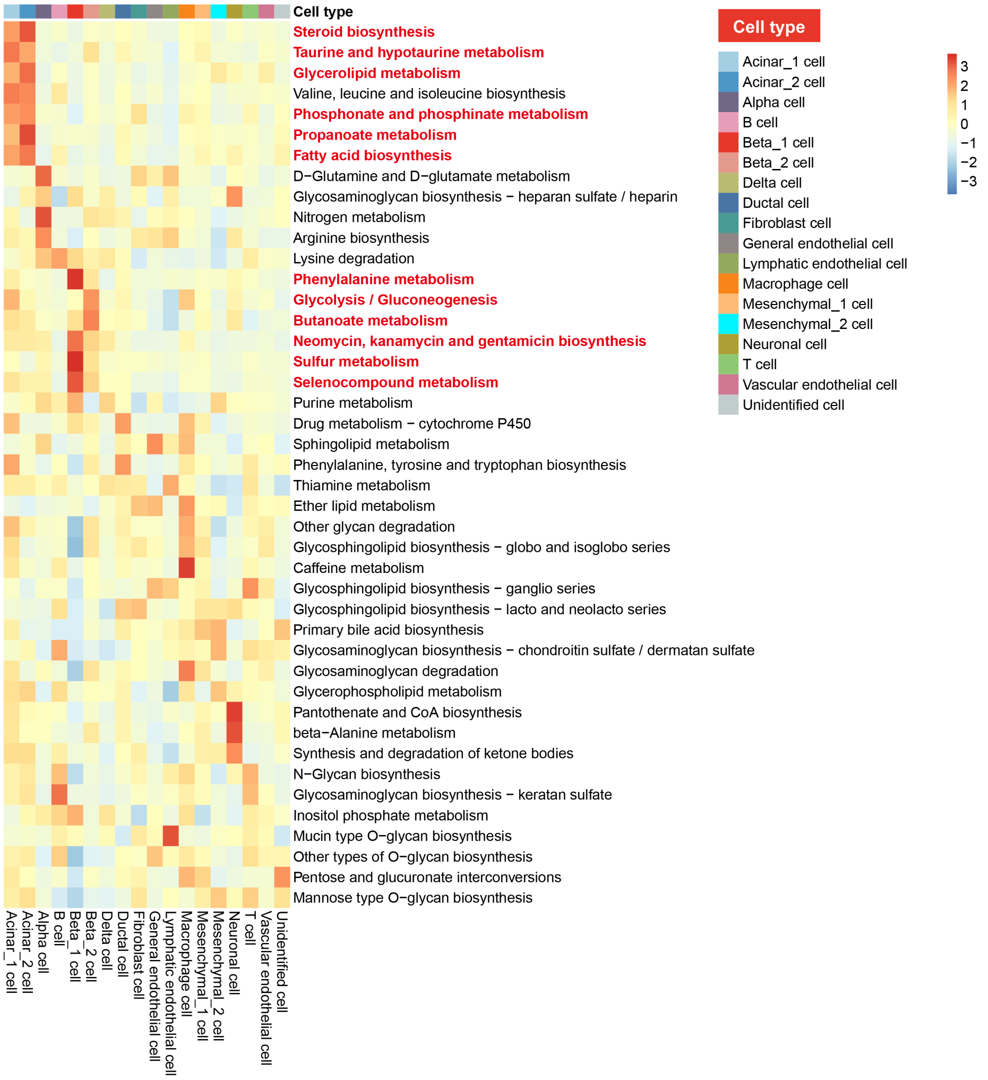

Figure S5. Heatmap showing the metabolic activity analysis of all clusters by scMetabolism R package, revealing the heterogeneity of metabolic pathways in the fetal pancreas (E18.5).

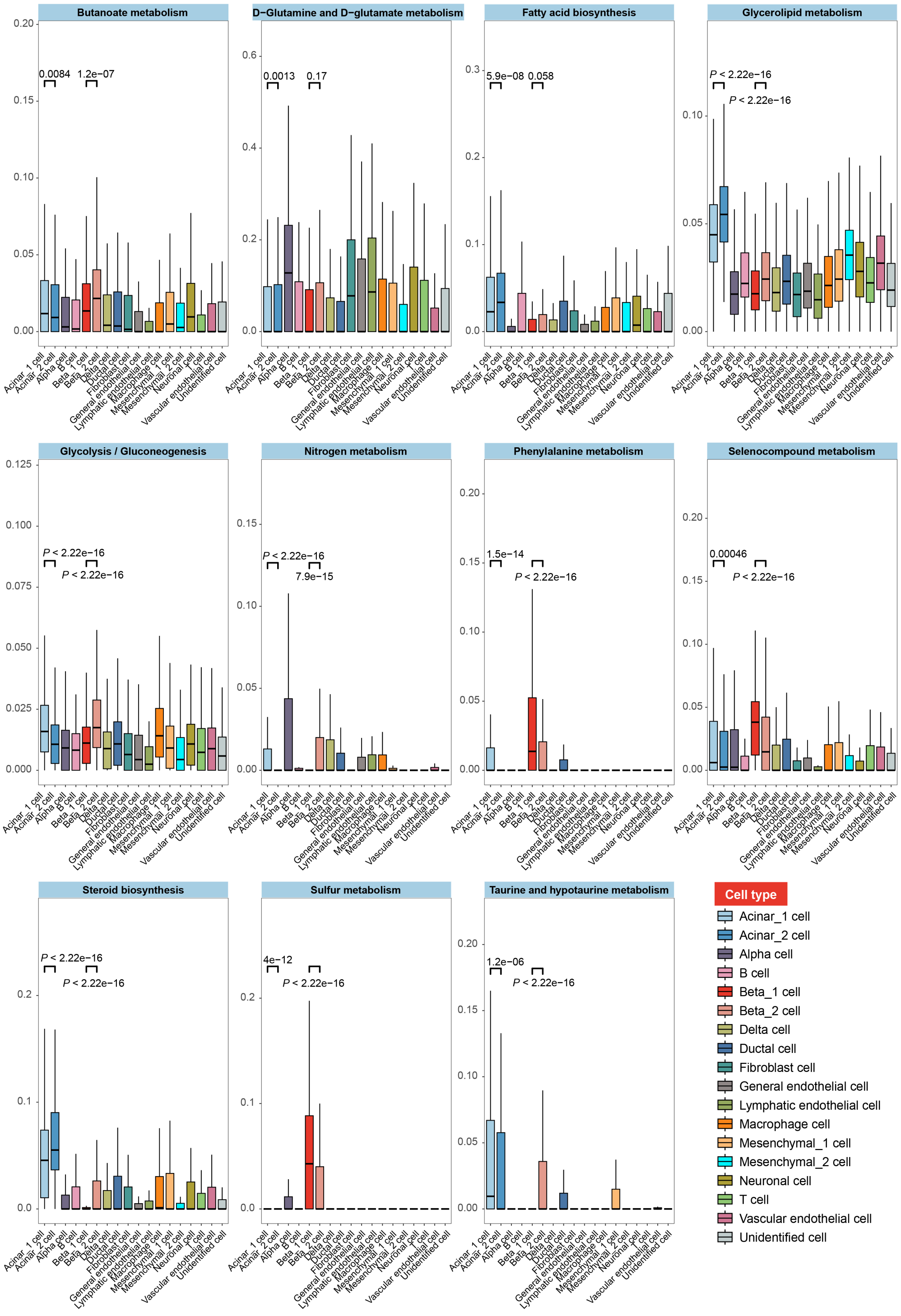

Figure S6. Further analysis of the metabolism pathways among acinar_1 cells, acinar_2 cells, beta_1 cells, and beta_2 cells in the fetal pancreas (E18.5) by scMetabolism R package.

The statistical difference was compared by wilcox.test.

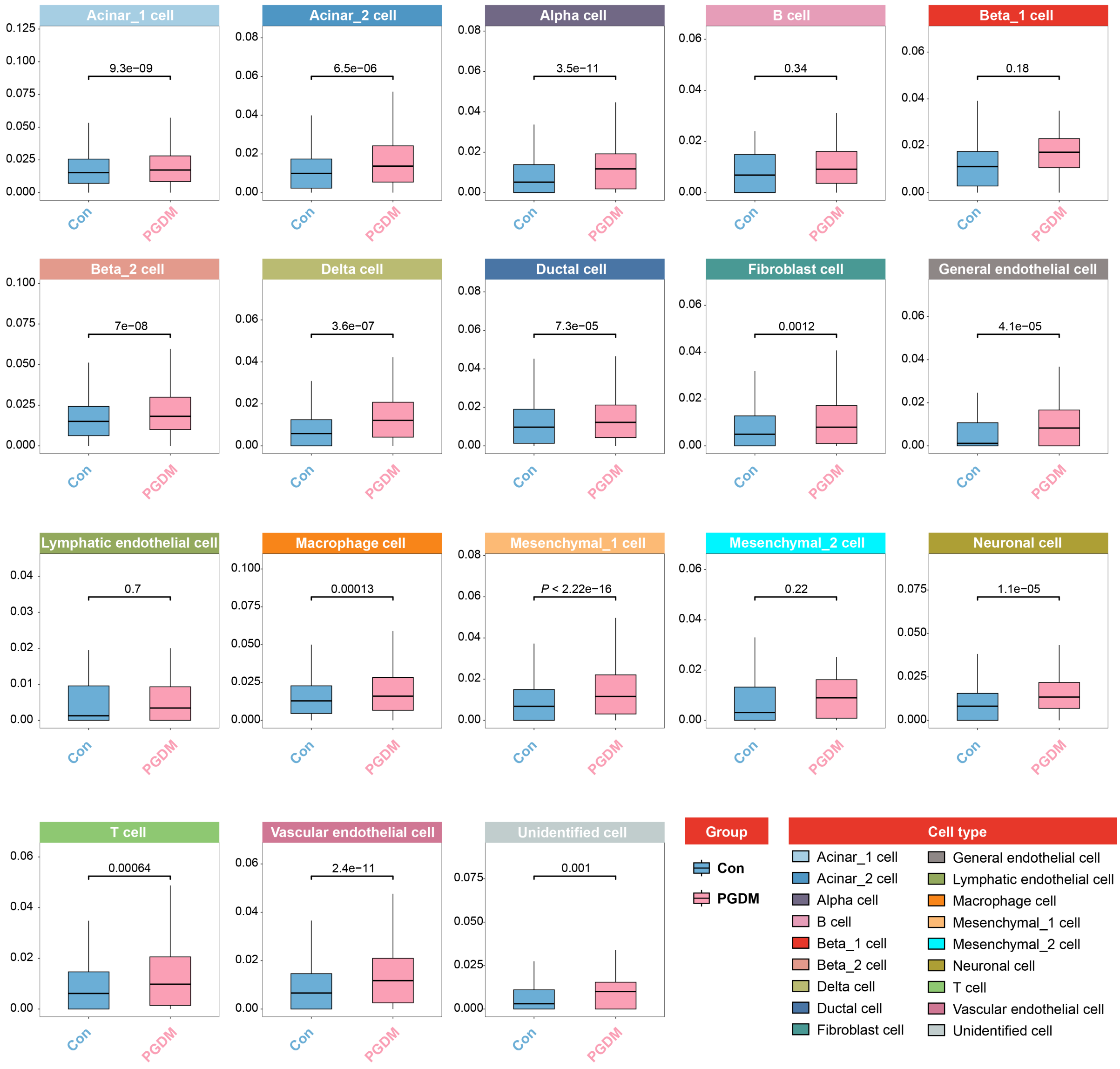

Figure S7. Comparison of the glycolysis/gluconeogenesis pathway in all clusters between PGDM (E18.5) and Con groups (E18.5).

The statistical difference was compared by wilcox.test.

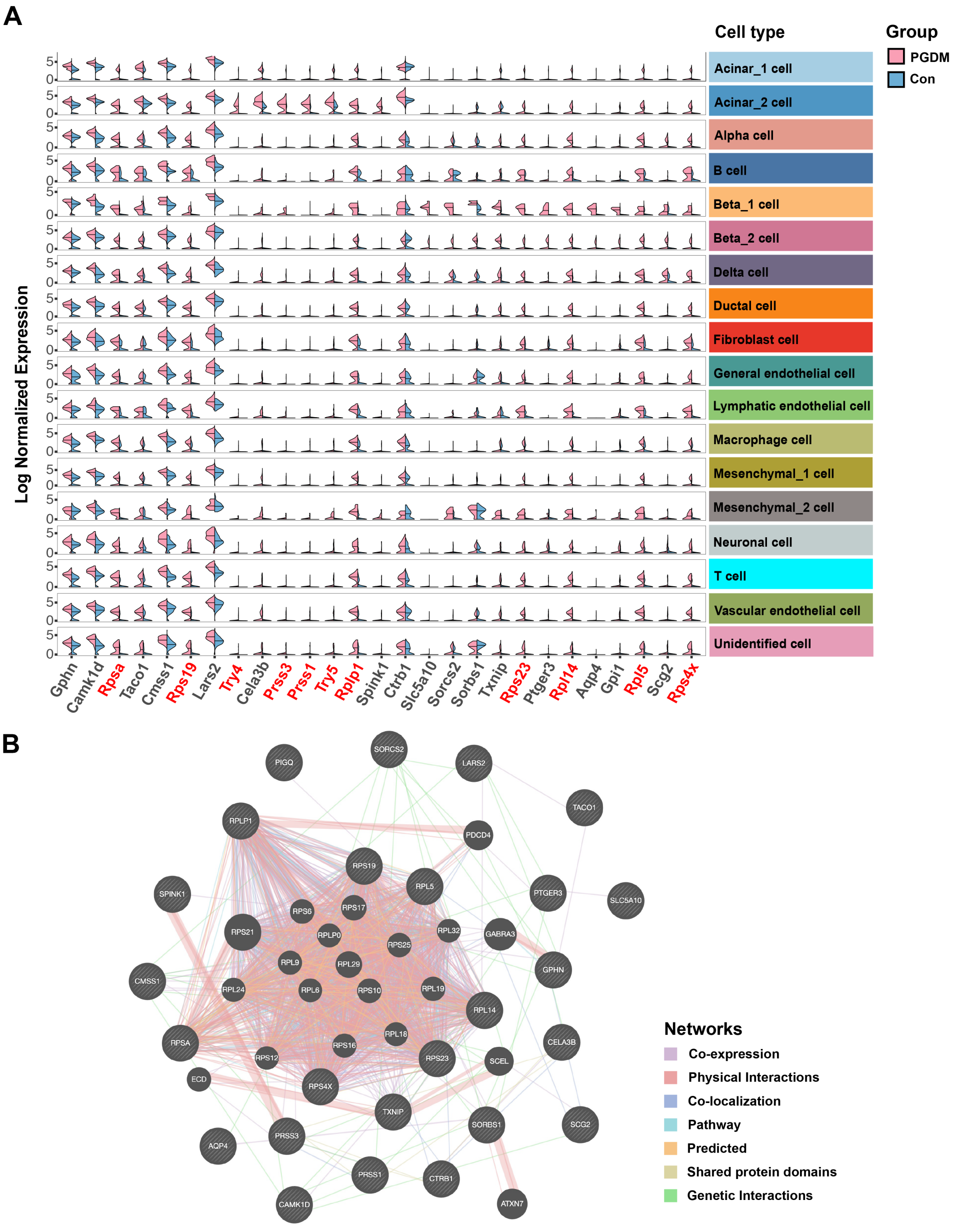

Figure S8. Gene expression analysis and PPI networks of select up-regulated DEGs at E18.5, related to Figure 2D.

(A) Violin plots showing expression levels of select up-regulated DEGs between PGDM and control (E18.5) groups across clusters. Clusters were indicated on the right side of the panel. The gene name was indicated below the violin plots. **(B)** GeneMANIA visualization of select up-regulated DEGs. PPI networks were generated using GeneMANIA (https://genemania.org/, accessed May 2024). Abbreviation: DEGs, differentially expressed genes; PPI, Protein-protein interaction.

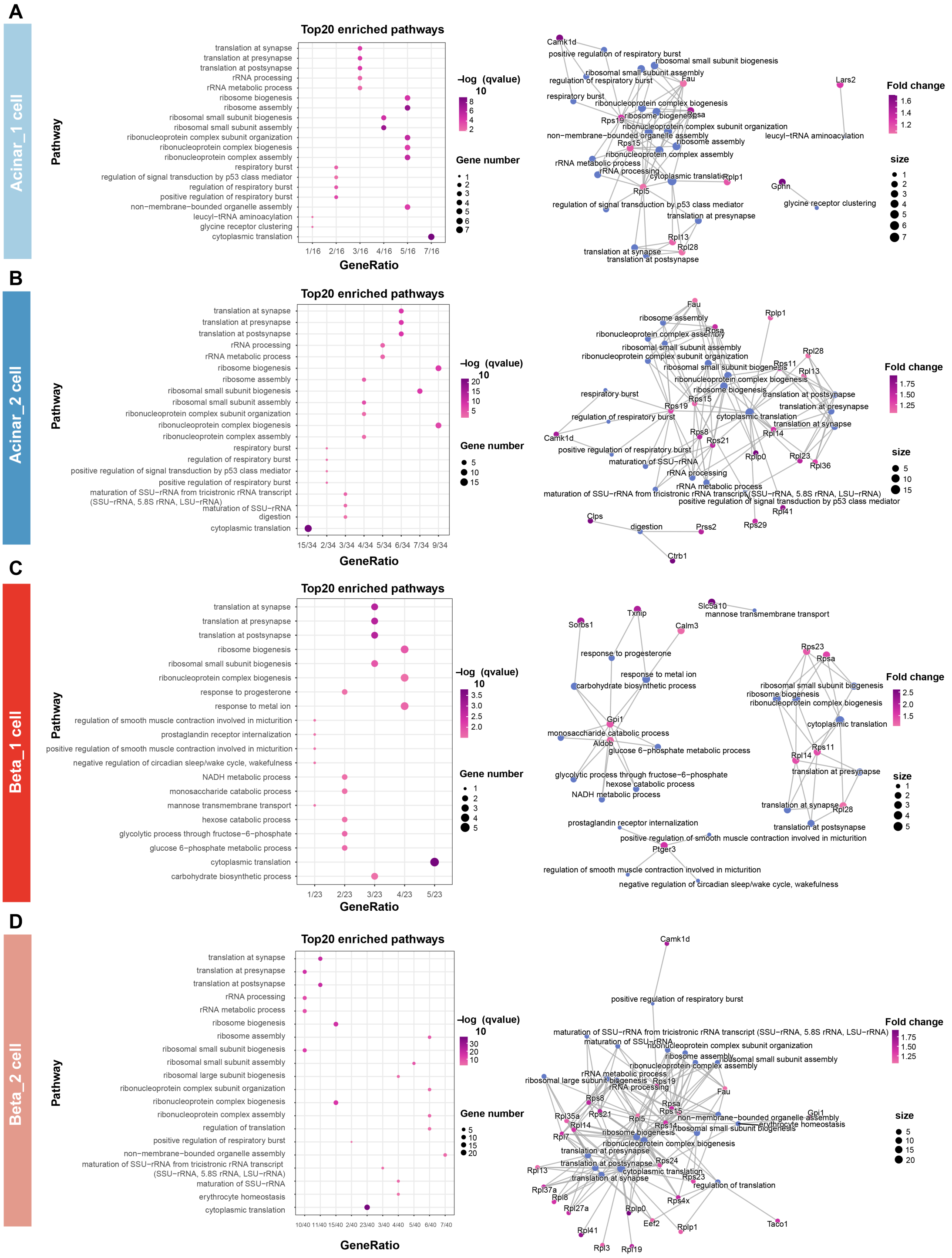

Figure S9. Gene ontology (GO) enrichment analysis of significantly upregulated genes between PGDM and control (E18.5) groups, related to Figure 2F.

(A-D) GO enrichment analysis of differentially upregulated genes (avg_log2FC > 1 and P_val_adj < 0.05) between PGDM and control (E18.5) groups across clusters (the acinar_1 cell, acinar_2 cell, beta_1 cell and beta_2 cell cluster). Bar plot (left panels) and cneplot (right panels) were used to visualize the GO terms.

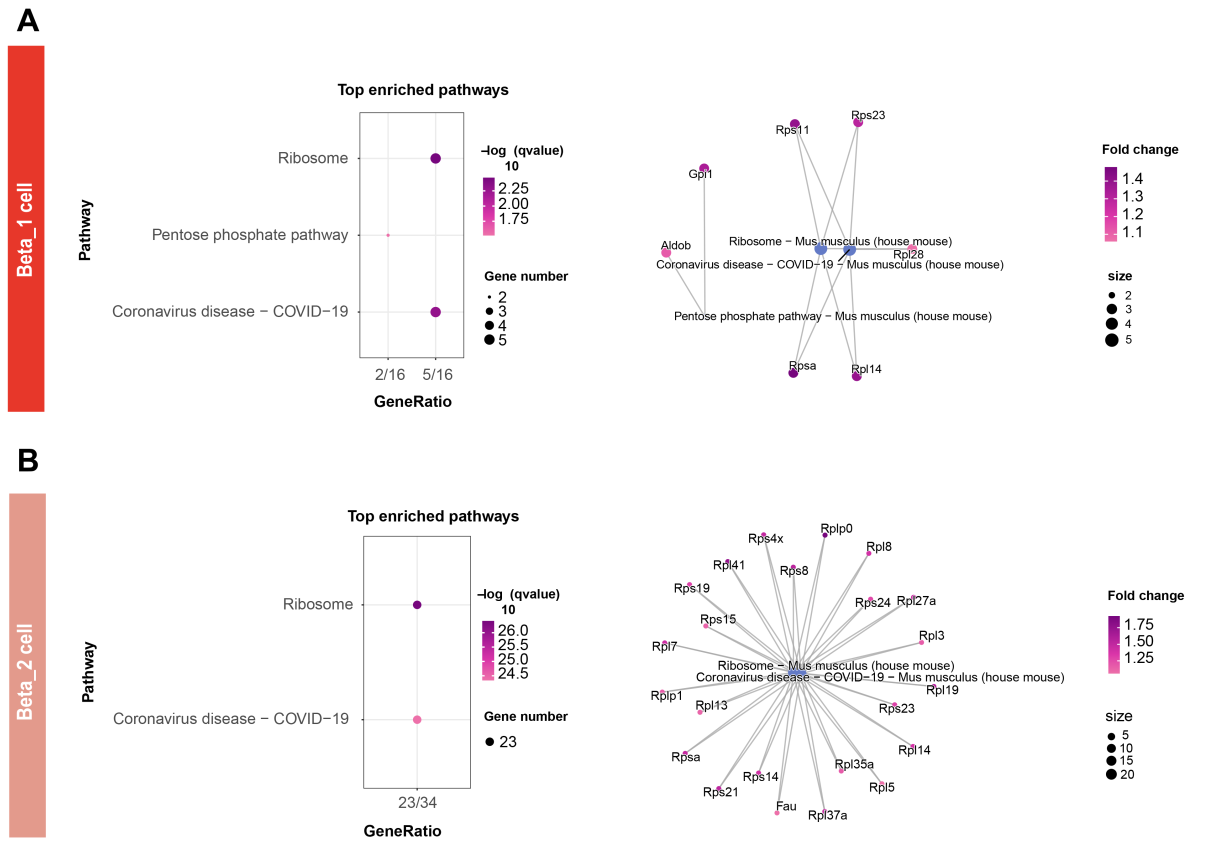

Figure S10. The kyoto encyclopedia of genes and genomes (KEGG) pathway analysis of significantly upregulated genes between PGDM and control (E18.5) groups, related to Figure 2F.

(A-B) KEGG pathway analysis of differentially upregulated genes (avg_log2FC > 1 and *P*_val_adj < 0.05) between PGDM and control (E18.5) groups across clusters (beta_1 cell and beta_2 cell cluster). Bar plot (left panels) and cneplot (right panels) were used to visualize the KEGG pathway terms.

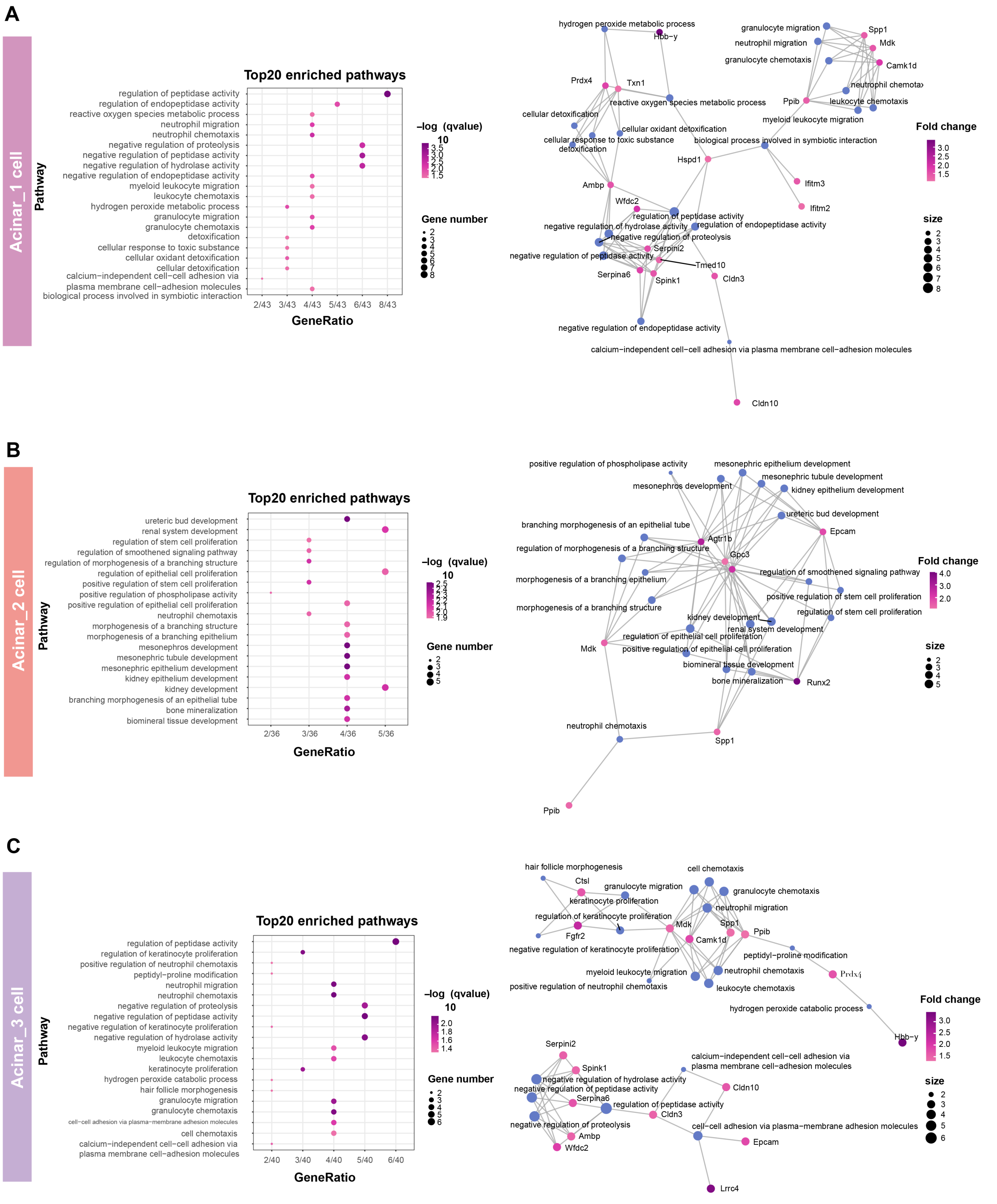

Figure S11. The GO enrichment analysis of significantly upregulated genes between PGDM and control (E16.5) groups, related to Figure 2F.

(A-C) GO enrichment analysis of differentially upregulated genes (avg_log2FC > 1 and P_val_adj < 0.05) between PGDM and control (E16.5) groups across clusters (the acinar_1 cell, acinar_2 cell and acinar_3 cell cluster). Bar plot (left panels) and cneplot (right panels) were used to visualize the GO terms.

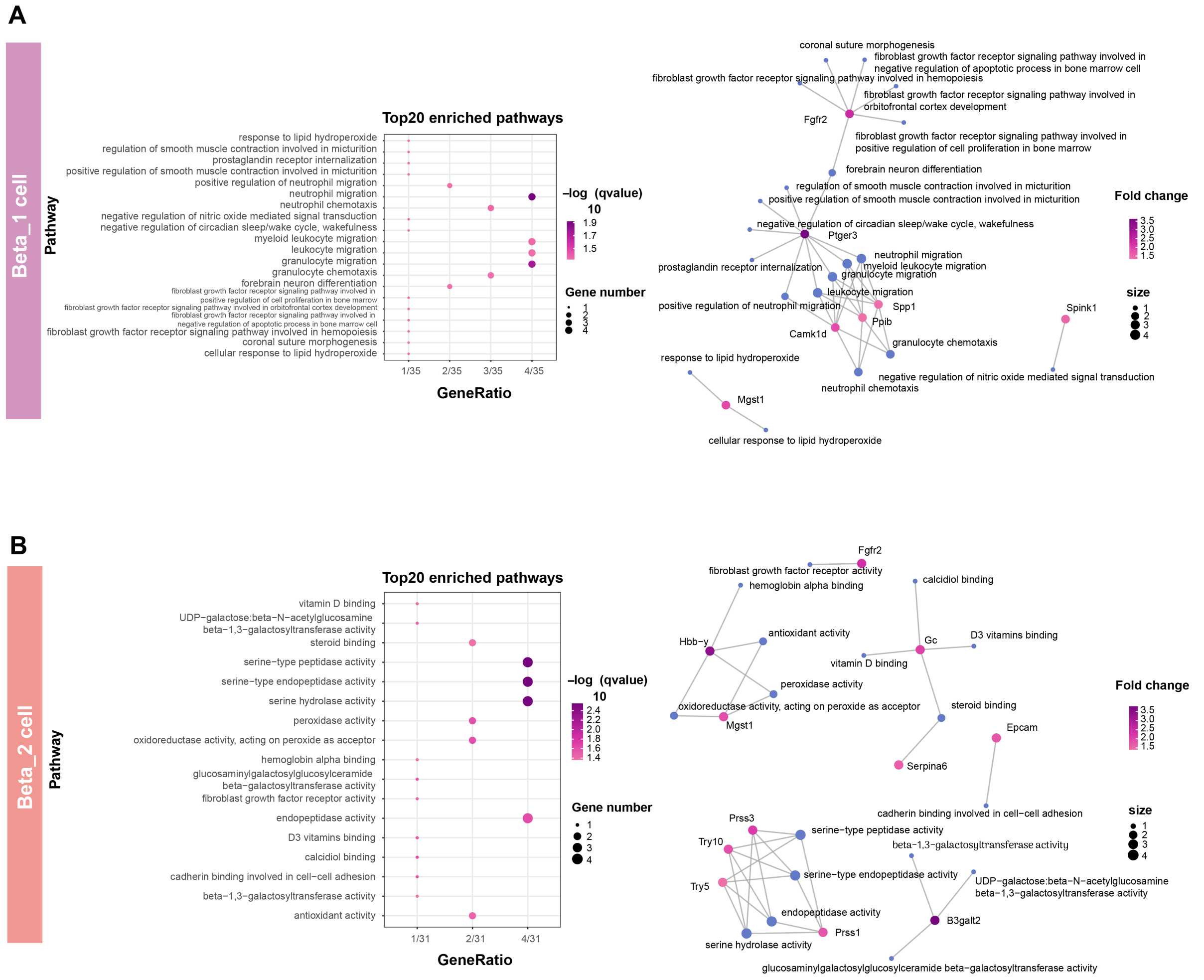

Figure S12. The GO enrichment analysis of significantly upregulated genes between PGDM and control (E16.5) groups, related to Figure 2F.

(A-B) GO enrichment analysis of differentially upregulated genes (avg_log2FC > 1 and *P*_val_adj < 0.05) between PGDM and control (E16.5) groups across clusters (the beta_1 cell and beta_2 cell cluster). Bar plot (left panels) and cneplot (right panels) were used to visualize the GO terms.

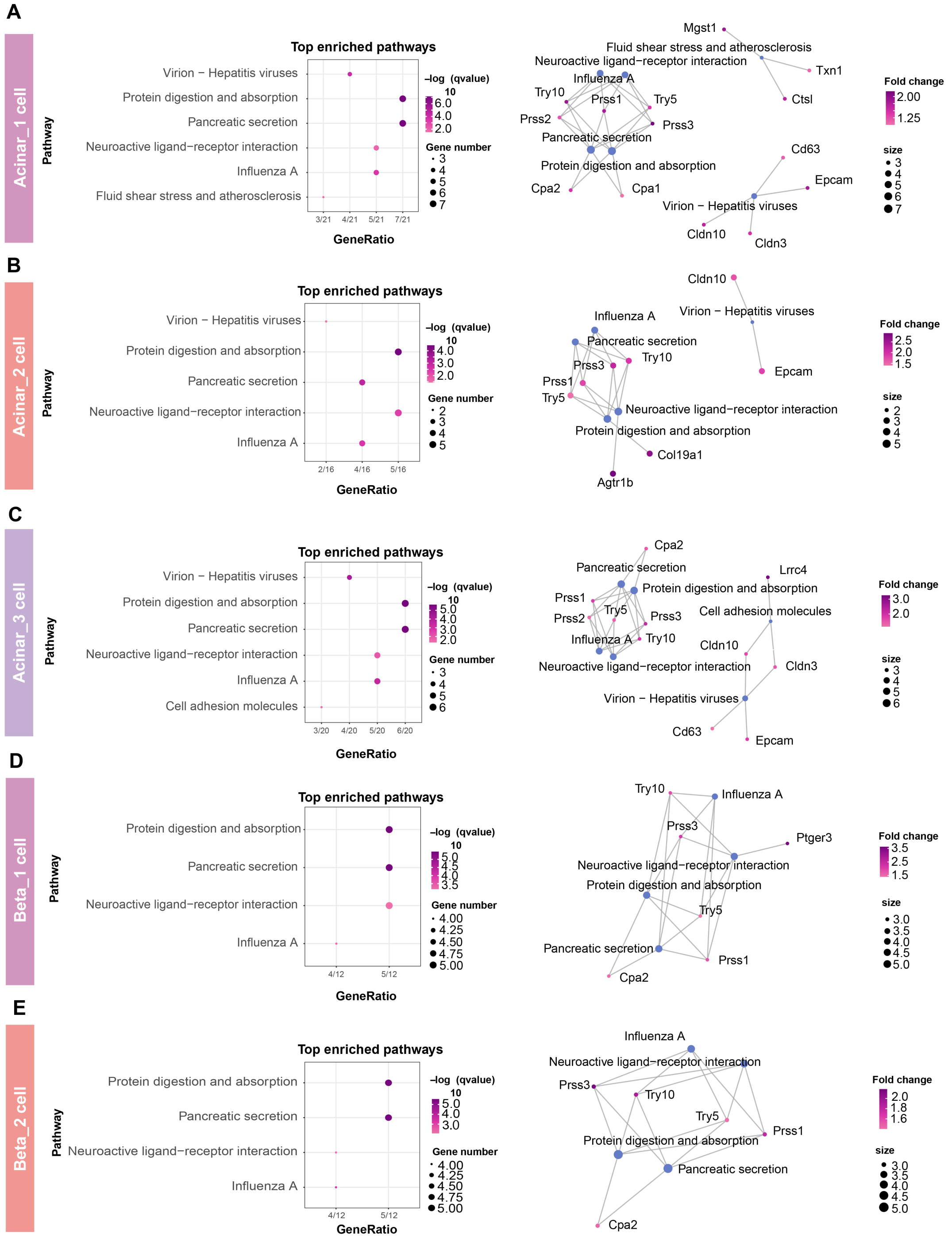

Figure S13. The KEGG pathway analysis of significantly upregulated genes between PGDM and control (E16.5) groups, related to Figure 2F.

(A-E) KEGG pathway analysis of differentially upregulated genes (avg_log2FC > 1 and *P*_val_adj < 0.05) between PGDM and control (E16.5) groups across clusters (the acinar_1 cell, acinar_2 cell, acinar_3 cell, beta_1 cell and beta_2 cell cluster). Bar plot (left panels) and cneplot (right panels) were used to visualize the KEGG pathway terms.

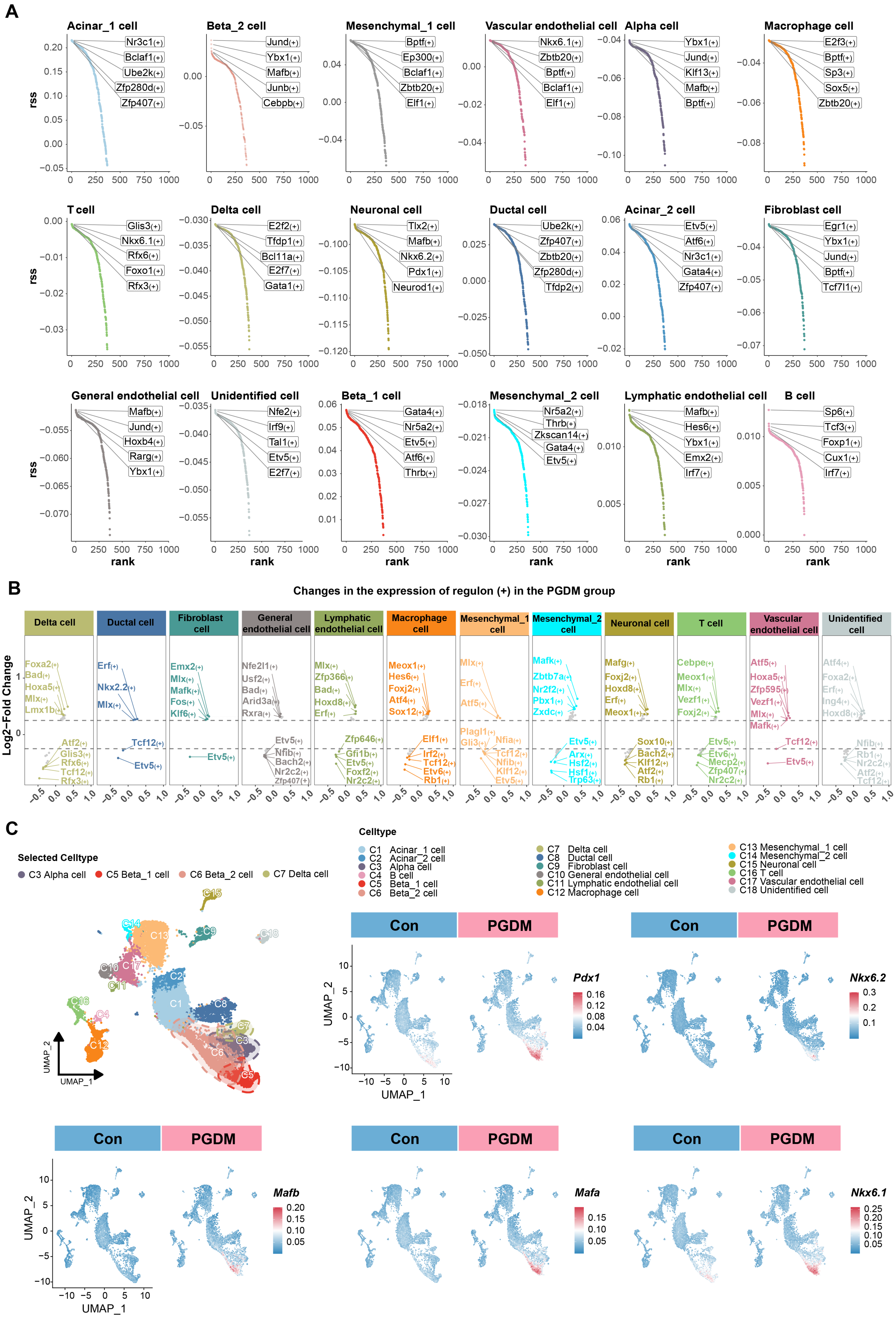
A
Acinar_1 cell
Nr3c1(+)
Bclaf1(+)
Ube2k(+)
Zfp280d(+)
Zfp407(+)
Beta_2 cell
Jund(+)
Ybx1(+)
Mafb(+)
Junb(+)
Cebpb(+)
Mesenchymal_1 cell
Bptf(+)
Ep300(+)
Bclaf1(+)
Zbtb20(+)
Elf1(+)
Vascular endothelial cell
Nkx6.1(+)
Zbtb20(+)
Bptf(+)
Bclaf1(+)
Elf1(+)
Alpha cell
Ybx1(+)
Jund(+)
Klf13(+)
Mafb(+)
Bptf(+)
Macrophage cell
E2f3(+)
Bptf(+)
Sp3(+)
Sox5(+)
Zbtb20(+)
T cell
Glis3(+)
Nkx6.1(+)
Rfx6(+)
Foxo1(+)
Rfx3(+)
Delta cell
E2f2(+)
Tfdp1(+)
Bcl11a(+)
E2f7(+)
Gata1(+)
Neuronal cell
Tlx2(+)
Mafb(+)
Nkx6.2(+)
Pdx1(+)
Neurod1(+)
Ductal cell
Ube2k(+)
Zfp407(+)
Zbtb20(+)
Zfp280d(+)
Tfdp2(+)
Acinar_2 cell
Etv5(+)
Atf6(+)
Nr3c1(+)
Gata4(+)
Zfp407(+)
Fibroblast cell
Egr1(+)
Ybx1(+)
Jund(+)
Bptf(+)
Tcf7l1(+)
General endothelial cell
Mafb(+)
Jund(+)
Hoxb4(+)
Rarg(+)
Ybx1(+)
Unidentified cell
Nfe2(+)
Irf9(+)
Tal1(+)
Etv5(+)
E2f7(+)
Beta_1 cell
Gata4(+)
Nr5a2(+)
Etv5(+)
Atf6(+)
Thrb(+)
Mesenchymal_2 cell
Nr5a2(+)
Thrb(+)
Zkscan14(+)
Gata4(+)
Etv5(+)
Lymphatic endothelial cell
Mafb(+)
Hes6(+)
Ybx1(+)
Emx2(+)
Irf7(+)
B cell
Sp6(+)
Tcf3(+)
Foxp1(+)
Cux1(+)
Irf7(+)
rss
rank
B
Changes in the expression of regulon (+) in the PGDM group
Log2-Fold Change
Delta cell
Ductal cell
Fibroblast cell
General endothelial cell
Lymphatic endothelial cell
Macrophage cell
Mesenchymal_1 cell
Mesenchymal_2 cell
Neuronal cell
T cell
Vascular endothelial cell
Unidentified cell
C
Selected Celltype
C3 Alpha cell
C5 Beta_1 cell
C6 Beta_2 cell
C7 Delta cell
Celltype
C1 Acinar_1 cell
C2 Acinar_2 cell
C3 Alpha cell
C4 B cell
C5 Beta_1 cell
C6 Beta_2 cell
C7 Delta cell
C8 Ductal cell
C9 Fibroblast cell
C10 General endothelial cell
C11 Lymphatic endothelial cell
C12 Macrophage cell
C13 Mesenchymal_1 cell
C14 Mesenchymal_2 cell
C15 Neuronal cell
C16 T cell
C17 Vascular endothelial cell
C18 Unidentified cell
UMAP_1
UMAP_2
Con
PGDM
Pdx1
Nkx6.2
Mafb
Mafa
Nkx6.1

Figure S14. Analysis of the cell type-specific regulons in the fetal pancreas (E18.5), related to Figure 3.

(A) Identification of cell type-specific regulons across clusters. Identified regulons were ranked based on the regulon specificity score (rss, y-axis). **(B)** SCENIC analysis revealed significant changes in the expression levels of regulons in the PGDM group within each cluster compared with the control group. **(C)** Feature plots depicting TF gene expression levels, including of *Pdx1*, *Nkx6.2*, *Mafb*, *Mafa*, and *Nkx6.1* between the PGDM and controls. Abbreviation: SCENIC, single-cell regulatory network inference and clustering; TF, transcription factor.

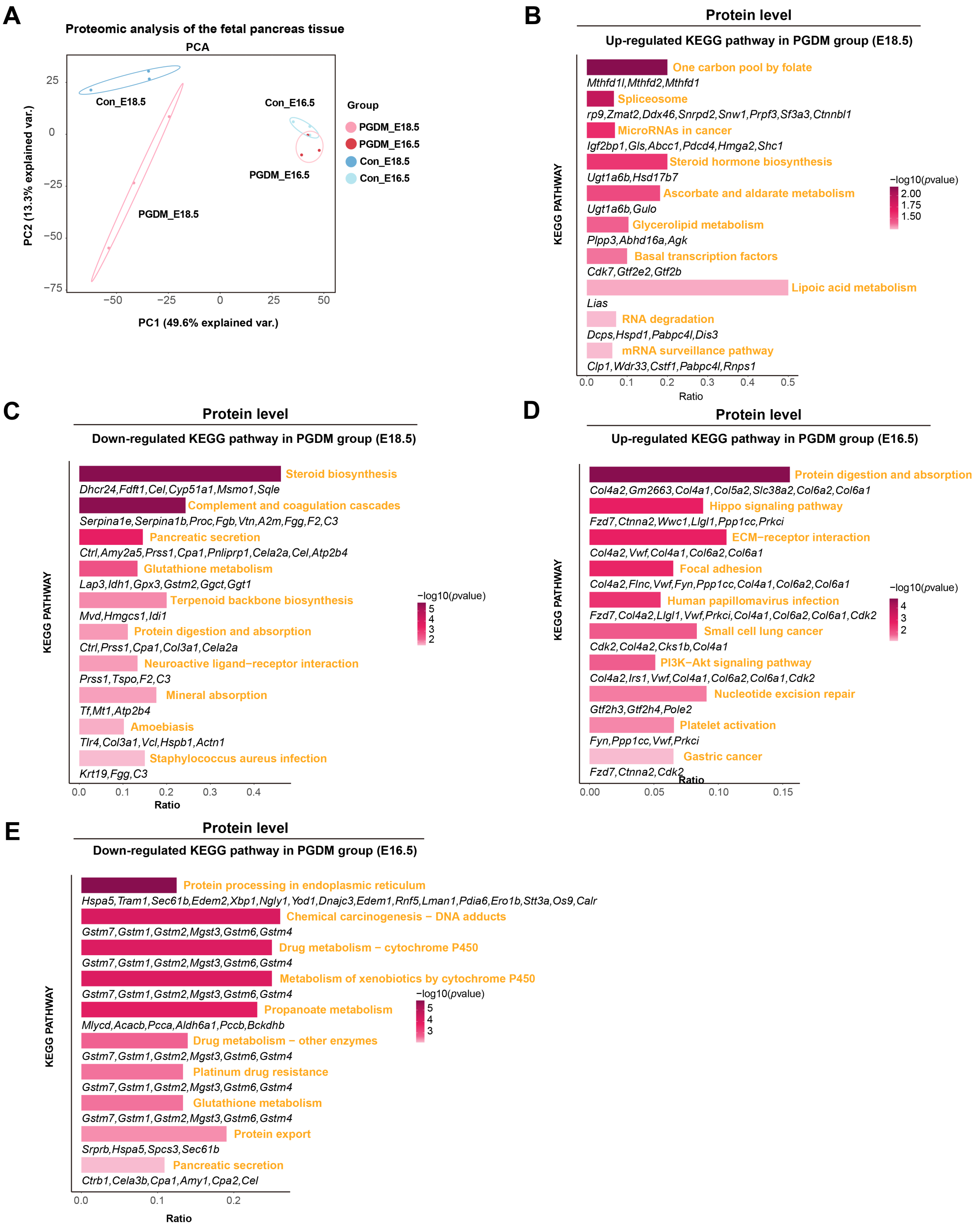

Figure S15. The KEGG pathway analysis of the significantly differentially expressed proteins between PGDM and control (E16.5 and E18.5) groups, related to Figure 3.

(A) The PCA of 12 samples used for proteomic analysis of the fetal pancreas tissue. **(B-E)** The KEGG pathway analysis of differentially expressed proteins (fold change cutoff = 1.2 and *P*-value < 0.05) between PGDM and control (E16.5 and E18.5) groups. The bar plot was used to visualize the KEGG pathway terms.

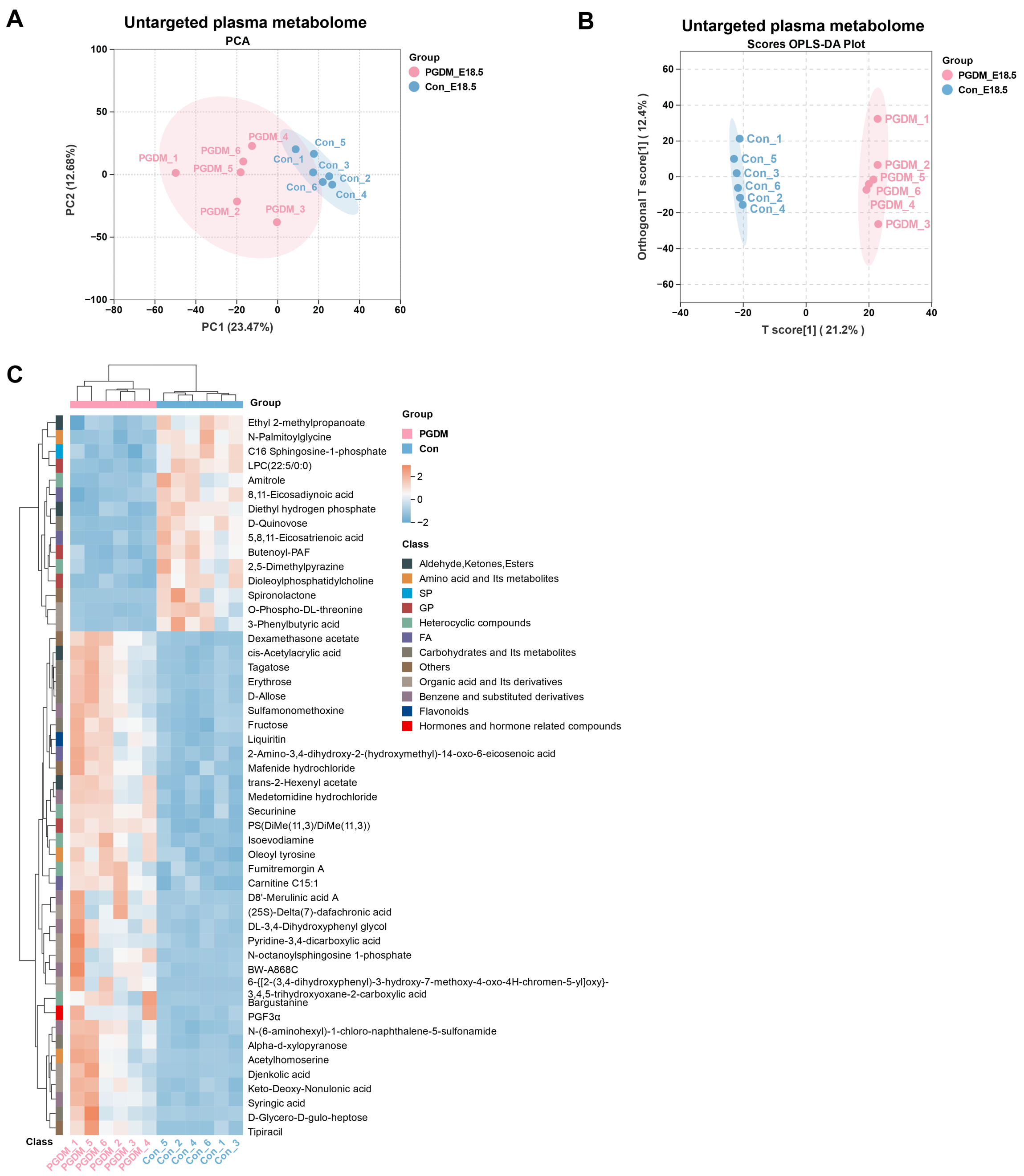

Figure S16. Metabolite analysis of plasma samples from 12 pregnant mice at E18.5.

(A) The PCA of 12 samples used for untargeted plasma metabolome. **(B)** OPLS-DA score plot of untargeted plasma metabolome. **(C)** The hierarchical clustering heatmap of the top 50 differential plasma metabolites of pregnant mice between PGDM and control (E18.5) groups. The differential metabolites were determined according to the VIP (VIP > 1) and *P*-value (*P*-value < 0.05, Student's t test). Abbreviation: OPLS-DA, orthogonal partial least-squared discriminant analysis; VIP, variable importance in projection.

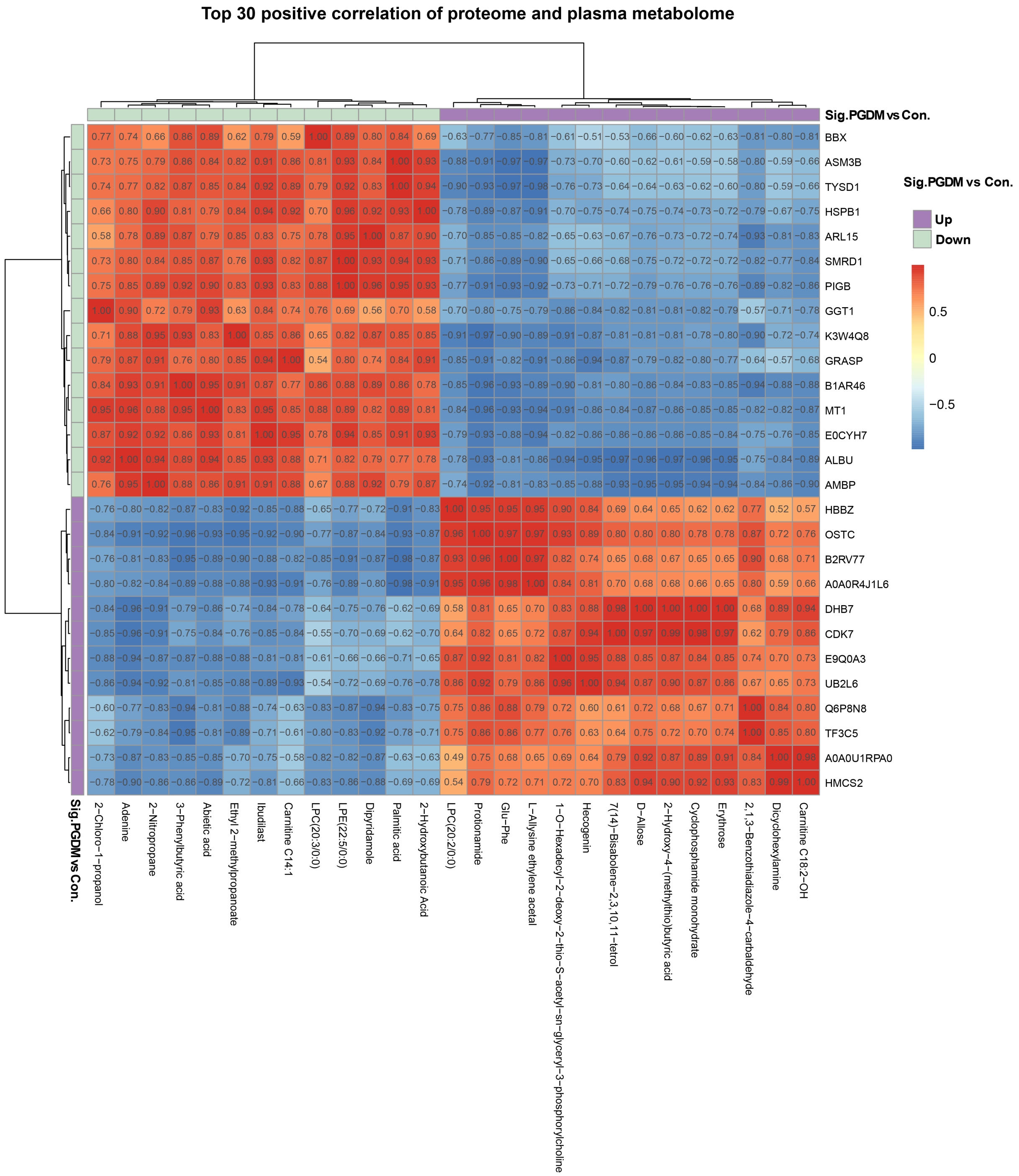

Figure S17. Correlation of differentially expressed proteins of fetal pancreas (E18.5) and differential plasma metabolites of pregnant mice (E18.5).

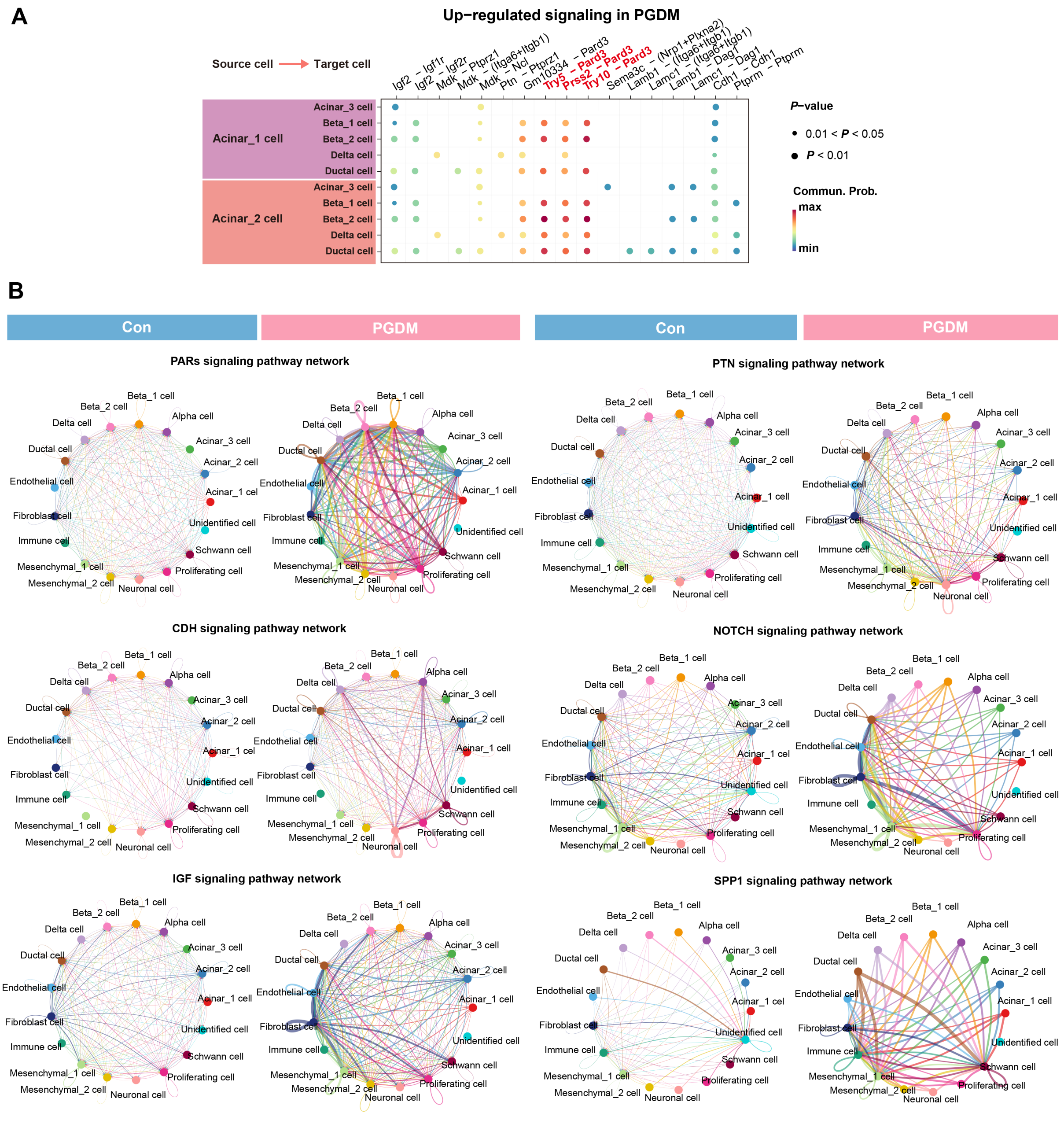

Figure S18. Cell–cell interaction analysis between acinar cells and multiple cell types in the presence of maternal PGDM (E16.5), related to Figure 4G.

(A) CellChat analysis showed that the ligand-receptor pairing signaling network based on snRNA-seq data was enhanced in the PGDM group (E16.5) compared to the control group (E16.5). **(B)** Circle network plots, derived from snRNA-seq data (E16.5), displaying the enhanced signaling pathways in the PGDM group compared to the control group, including PARs, PTN, CDH, NOTCH, IGF, and SPP1 signaling pathway. Abbreviations: PARs, protease-activated receptors; PTN, pleiotrophin; CDH, cadherin; IGF, insulin-like growth factor; SPP1, secreted phosphoprotein 1.

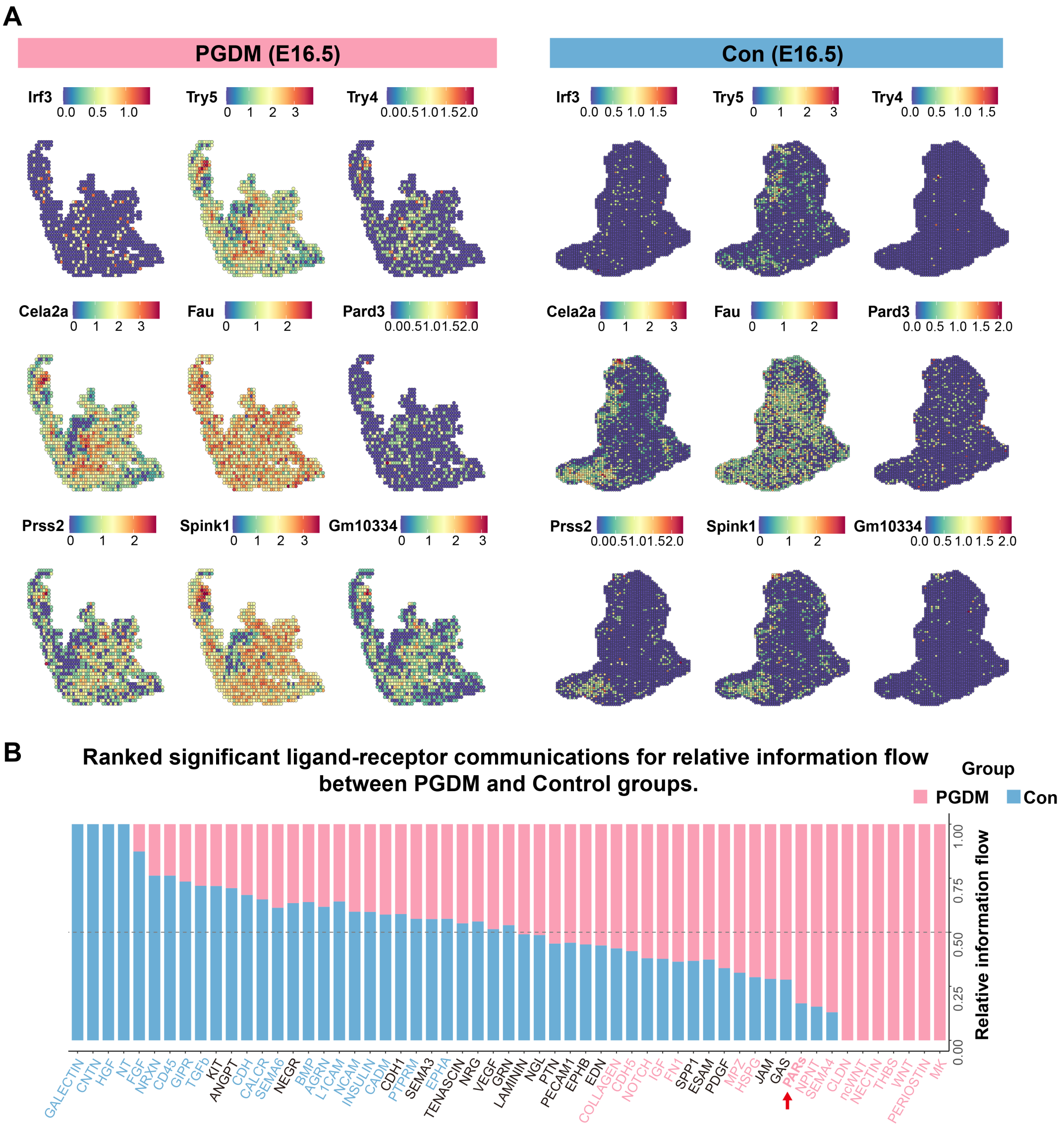

Figure S19. Cellular communication features between acinar cells and multiple cell types, related to Figure 4.

(A) ST data based on spots (bin 50, 50 × 50 nanoballs aggregated) showing the gene expression level of *Irf3, Try5, Try4, Cela2a, Fau, Pard3, Prss2, Spink1, and Gm10334* in the PGDM (E16.5) and control (E16.5) group. **(B)** Comparison of the overall information flow of each signaling pathway between the PGDM (E18.5) and control (E18.5) group**.** Significantly enriched signaling pathways in the PGDM group were colored in red text, and signaling pathways with blue text were significantly enriched in the control group.

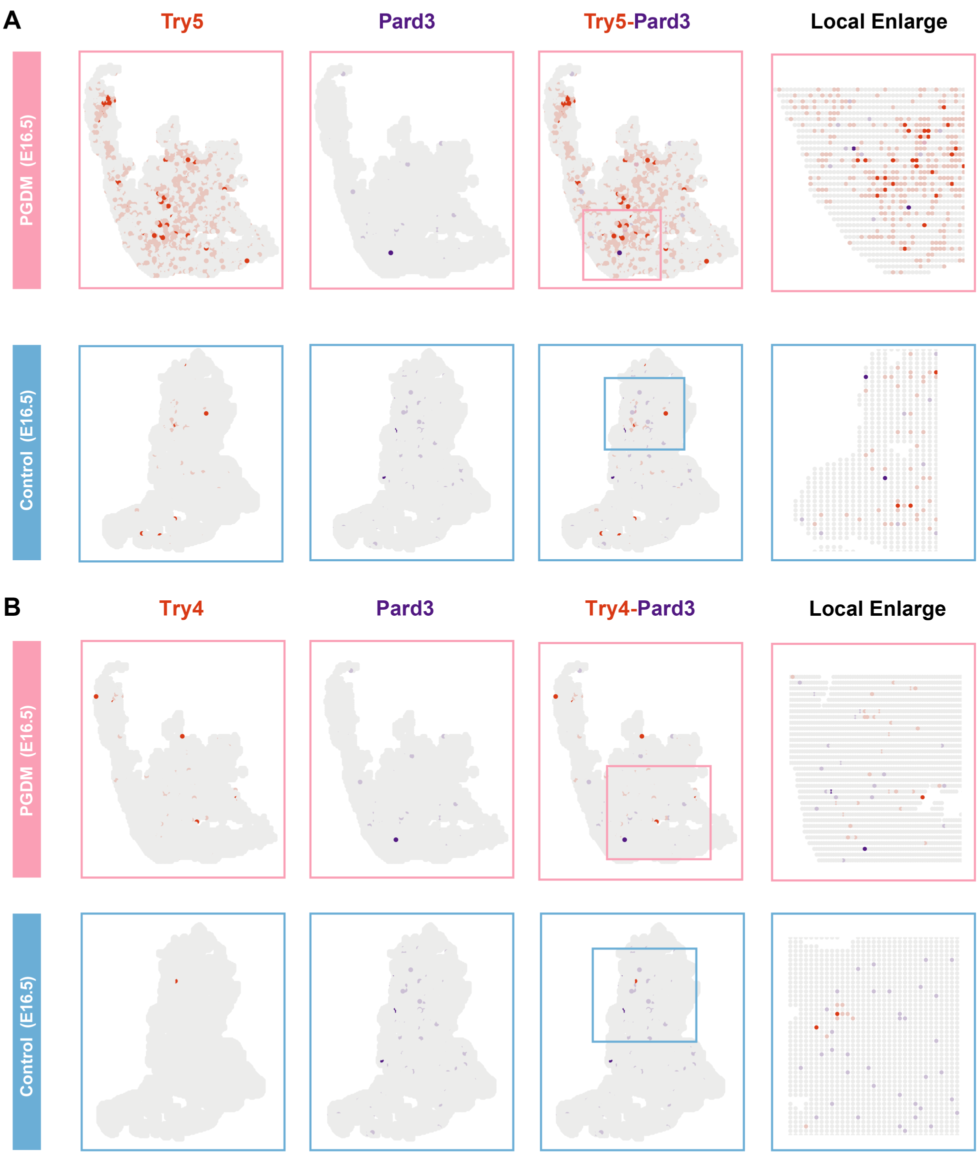

Figure S20. Spatial distribution analysis of Try5, Try4 and Pard3 based on E16.5 ST data, related to Figure 4.

(A-B) The ST data based on spots (bin 20, 20 × 20 nanoballs aggregated) showing Try5 (red) and Try4 (red) were located in close proximity to the Pard3 (purple). Abbreviations: ST, spatial transcriptomics.

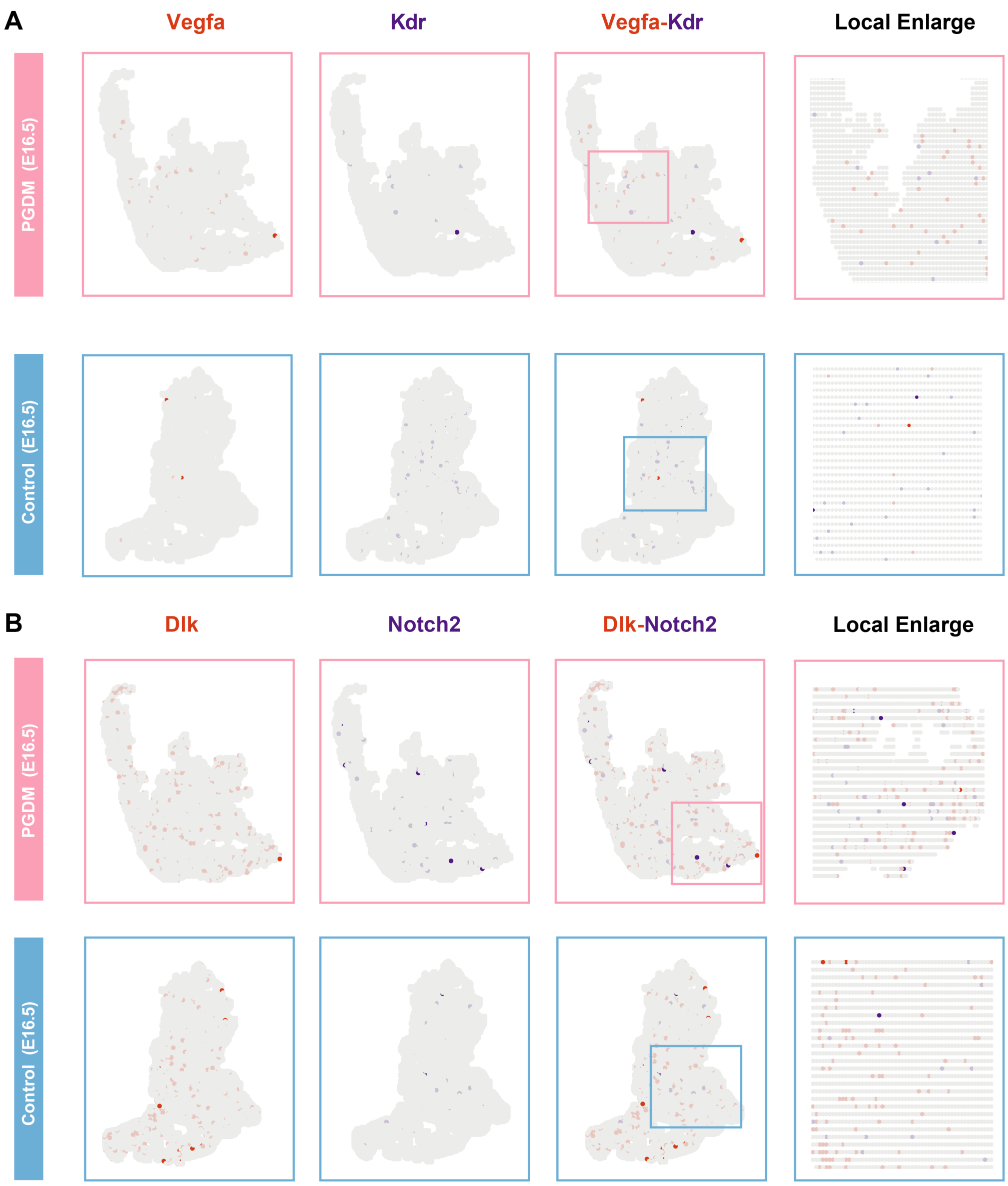

Figure S21. Spatial distribution analysis of Vegfa, Kdr, Dlk and Notch2 based on E16.5 ST data, related to Figure 5.

(A) The ST data based on spots (bin 20, 20 × 20 nanoballs aggregated) showing Vegfa (red) was located in close proximity to the Kdr (purple). **(B)** The ST data based on spots (bin 20, 20 × 20 nanoballs aggregated) showing Dlk (red) was located in close proximity to the Notch2 (purple).

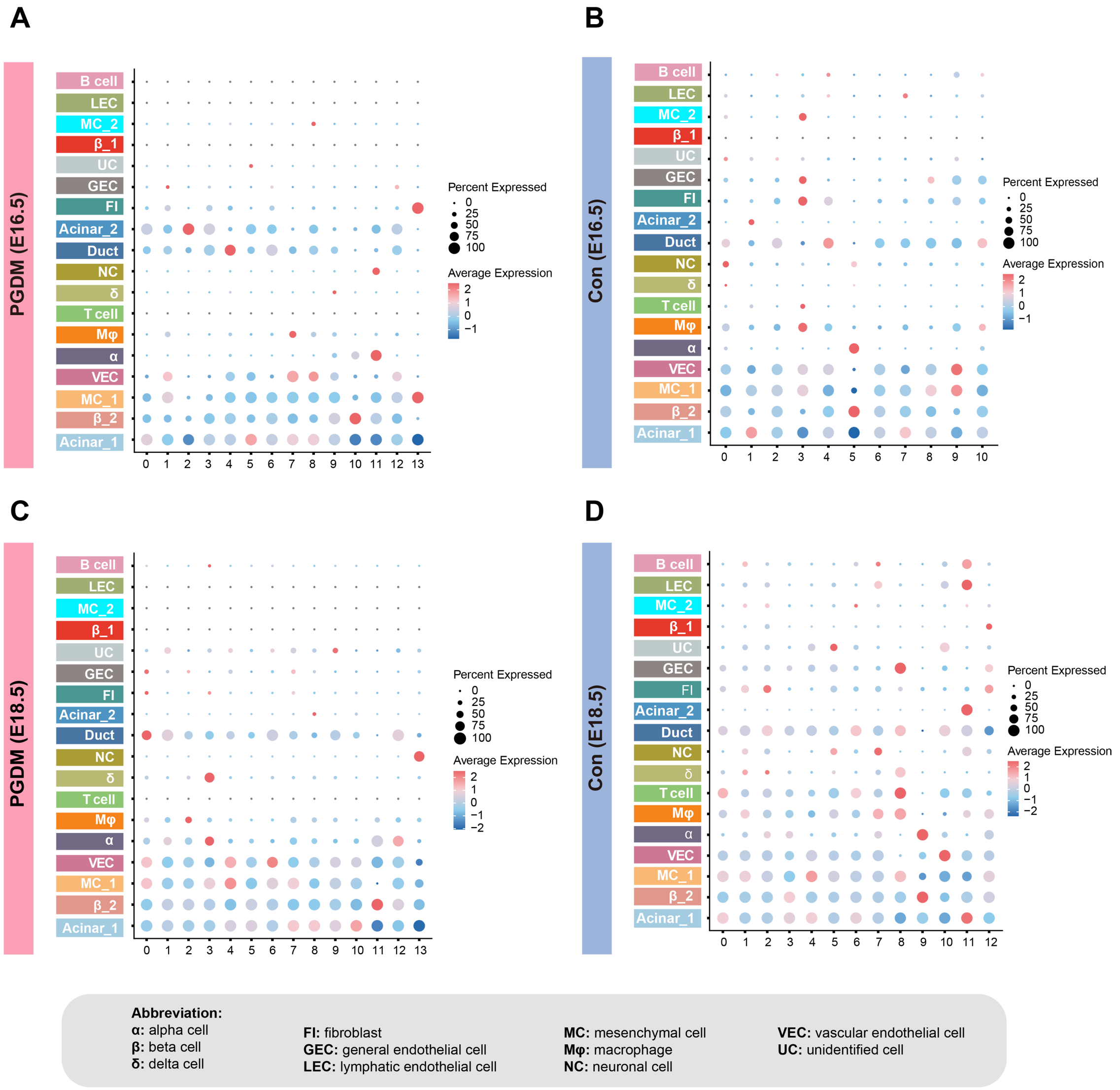

Figure S22. Cell neighborhood analysis, related to Figure 6.

(A-D) Integration of spatial and single-nucleus transcriptome information in the PGDM group (A) and control group (B) at E16.5, and the PGDM group (C) and control group (D) at E18.5. Cell types in stereo-seq chips were annotated with snRNA-seq data by using "TransferData" function. The x-coordinate axis represented cell clusters identified by ST analysis and the y-coordinate axis represented cell clusters identified by snRNA-seq analysis.

Table S1. Cluster annotations based on top differentially expressed genes in single nucleus RNA sequencing analysis (E18.5).

Clusters (E18.5)	Marker genes (E18.5)
Acinar_1 cell	*Rbpjl* [1] and *Sel1l* [2]
Acinar_2 cell	*Cpb1* [3], *Amy2a1* [4], and *Pnliprp1* [5]
Alpha cell	*Gcg* [6] and *Nxph1* [7]
B cell	*Blnk* [8], *Pou2af1* [9], and *Slamf6* [10]
Beta_1 cell	*Trpm3* [11], *Kcnip1* [12], and *Robo2* [13]
Beta_2 cell	*Mlxipl* [14] and *Pax6* [15]
Delta cell	*Sst* [6a]
Ductal cell	*Bicc1* [16] and *Erbb4* [17]
Fibroblast cell	*Sulf1* [18] and *Wt1* [19]
General endothelial cell	*Vwf* and *Pecam1* [20]
Lymphatic endothelial cell	*Flt4* [21] and *Mmrn1* [22]
Macrophage cell	*F13a1* [23] and *Mrc1* [24]
Mesenchymal_1 cell	*Col3a1* [25], *Vcan* [26], and *Adam12* [27]
Mesenchymal_2 cell	*Acta2*, *Actg2* [28], and *Col8a1* [29]
Neuronal cell	*Nrg3* [30], *Syt1* [31], and *Nrxn1* [32]
T cell	*Il7r* [33], *Il18r1* [34], and *Itga4* [35]
Vascular endothelial cell	*Flt1* [36] and *Eng* [37]

Table S2. Cluster annotations based on top differentially expressed genes in single nucleus RNA sequencing analysis (E16.5).

Clusters (E16.5)	Marker genes (E16.5)
Acinar_1 cell	*Try5* [38], *Try10* and *Prss1*[39]
Acinar_2 cell	*Rbpjl* [1] and *Esrrg* [40]
Acinar_3 cell	*Rap1gap* [41]
Alpha cell	*Nxph1* [7] and *Wnk3* [7]
Beta_1 cell	*Mlxipl* [14] and *Tmem163* [42]
Beta_2 cell	*Kcnb2* [43] and *Rfx6* [44]
Delta cell	*Sst* [6a]
Ductal cell	*Bicc1* [16] and *Pkhd1* [45]
Endothelial cell	*Flt1* [36], *Pecam1* [20], *Ptprm* [20] and *Eng* [46]
Fibroblast cell	*Sulf1* [18], *Wt1* [19] and *Bnc2* [47]
Immune cell	*F13a1* [48] and *Ptprc* [49]
Mesenchymal_1 cell	*Slit2* [50] and *Epha3* [51]
Mesenchymal_2 cell	*Tshz2* [52] and *Pde3a* [53]
Neuronal cell	*Nrg3* [30], *Syt1* [31], and *Nrxn1* [32]
Proliferating cell	*Top2a* [54] and *Cenpp* [55]
Schwann cell	*Slc35f1* [56], *Csmd1* [56] and *Cdh19* [25]

Table S3. The data quality of single nucleus RNA sequencing.

Sample name	Estimated number of cells	Mean reads per cell	Median genes per cell	Number of reads	Total genes detected	Median UMI counts per cell
PGDM-(NO.1-3) (E16.5)	13832	30059	1213	415,774,567	22232	1880
Control-(NO.4-5) (E16.5)	12117	31629	1314	383,248,596	21750	2051
Control-(NO.6-7) (E16.5)	13687	27114	1548	371,112,138	21738	2508
PGDM-(NO.8-9) (E18.5)	4515	78978	648	356,584,488	21085	1077
PGDM-(NO.10-11) (E18.5)	6056	62983	724	381,423,828	22688	1310
Control-(NO.12-13) (E18.5)	4931	64906	807	320,053,230	22995	1180
Control-(NO.14-15) (E18.5)	7889	53477	838	421,881,113	23633	1265

Table S4. The data quality of stereo-seq chips.

Sample name	Total reads	Unique reads	Clean reads	Genes under tissue	Mean MID per bin200	Mean gene type per bin200	Mean MID per bin50	Mean gene type per bin50
PGDM-(NO.16) (E16.5)	432,303,946	67,947,252	82.6%	21632	39142	6701	3087	1498
Control-(NO.17) (E16.5)	1,383,198,944	49,025,248	88.9%	21149	36358	4673	2880	967
PGDM-(NO.18) (E18.5)	272,821,647	13,054,552	78.3%	26276	9998	2078	769	277
PGDM-(NO.19) (E18.5)	1,148,147,467	11,711,868	81.7%	21780	12857	1636	1031	233

Control-(NO.20) (E18.5)	262,443,323	9,220,156	66.4%	27782	4958	2163	412	243
Control-(NO.21) (E18.5)	1,078,043,868	18,045,573	82.7%	25535	8612	1699	684	224

Supplementary References

[1] a) A. Bastidas-Ponce, K. Scheibner, H. Lickert, M. Bakhti, *Development* **2017**, *144* (16), 2873; b) T. Masui, G. H. Swift, T. Deering, C. Shen, W. S. Coats, Q. Long, H. P. Elsässer, M. A. Magnuson, R. J. MacDonald, *Gastroenterology* **2010**, *139* (1), 270.

[2] M. Cattaneo, S. Orlandini, S. Beghelli, P. S. Moore, C. Sorio, A. Bonora, C. Bassi, G. Talamini, G. Zamboni, R. Orlandi, S. Ménard, L. R. Bernardi, I. Biunno, A. Scarpa, *Oncogene* **2003**, *22* (41), 6359.

[3] K. Tamura, J. Yu, T. Hata, M. Suenaga, K. Shindo, T. Abe, A. MacGregor-Das, M. Borges, C. L. Wolfgang, M. J. Weiss, J. He, M. I. Canto, G. M. Petersen, S. Gallinger, S. Syngal, R. E. Brand, A. Rustgi, S. H. Olson, E. Stoffel, M. L. Cote, G. Zogopoulos, J. B. Potash, F. S. Goes, R. W. McCombie, P. P. Zandi, M. Pirooznia, M. Kramer, J. Parla, J. R. Eshleman, N. J. Roberts, R. H. Hruban, A. P. Klein, M. Goggins, *Proc Natl Acad Sci U S A* **2018**, *115* (18), 4767.

[4] N. Niu, P. Lu, Y. Yang, R. He, L. Zhang, J. Shi, J. Wu, M. Yang, Z. G. Zhang, L. W. Wang, W. Q. Gao, A. Habtezion, G. G. Xiao, Y. Sun, L. Li, J. Xue, *Gut* **2020**, *69* (4), 715.

[5] S. A. Campbell, C. L. McDonald, N. A. J. Krentz, F. C. Lynn, B. G. Hoffman, *Cell Rep* **2019**, *28* (7), 1830.

[6] a) T. Manea, J. K. Nelson, C. M. Garrone, K. Hansson, I. Evans, A. Behrens, R. Sancho, *Nat Commun* **2023**, *14* (1), 2457; b) D. Oropeza, P. L. Herrera, *Trends in Cell Biology* **2024**, *34* (3), 180.

[7] K. Hrovatin, A. Bastidas-Ponce, M. Bakhti, L. Zappia, M. Büttner, C. Salinno, M. Sterr, A. Böttcher, A. Migliorini, H. Lickert, F. J. Theis, *Nat Metab* **2023**, *5* (9), 1615.

[8] R. Pappu, A. M. Cheng, B. Li, Q. Gong, C. Chiu, N. Griffin, M. White, B. P. Sleckman, A. C. Chan, *Science* **1999**, *286* (5446), 1949.

[9] C. Zhao, J. Inoue, I. Imoto, T. Otsuki, S. Iida, R. Ueda, J. Inazawa, *Oncogene* **2008**, *27* (1), 63.

[10] N. Wang, M. Keszei, P. Halibozek, B. Yigit, P. Engel, C. Terhorst, *Clin Immunol* **2016**, *173*, 19.

[11] R. B. Kang, Y. Li, C. Rosselot, T. Zhang, M. Siddiq, P. Rajbhandari, A. F. Stewart, D. K. Scott, A. Garcia-Ocana, G. Lu, *Genome Med* **2023**, *15* (1), 30.

[12] H. S. Lee, S. Moon, J. H. Yun, M. Lee, M. Y. Hwang, Y. J. Kim, B. G. Han, J. M. Kim, B. J. Kim, *Genomics* **2014**, *104* (2), 113.

[13] Y. H. Yang, J. E. Manning Fox, K. L. Zhang, P. E. MacDonald, J. D. Johnson, *Proc Natl Acad Sci U S A* **2013**, *110* (41), 16480.

[14] a) M. A. Scavuzzo, M. C. Hill, J. Chmielowiec, D. Yang, J. Teaw, K. Sheng, Y. Kong, M. Bettini, C. Zong, J. F. Martin, M. Borowiak, *Nat Commun* **2018**, *9* (1), 3356; b) O. S. de la, X. Yao, S. Chang, Z. Liu, J. B. Sneddon, *Mol Metab* **2023**, *73*, 101735.

[15] W. Y. So, Y. Liao, W. N. Liu, G. A. Rutter, W. Han, *EMBO Mol Med* **2023**, *15* (12), e17928.

[16] C. A. Gonçalves, M. Larsen, S. Jung, J. Stratmann, A. Nakamura, M. Leuschner, L. Hersemann, R. Keshara, S. Perlman, L. Lundvall, L. L. Thuesen, K. J. Hare, I. Amit, A. Jørgensen, Y. H. Kim, A. Del Sol, A. Grapin-Botton, *Nat Commun* **2021**, *12* (1), 3144.

[17] C. Rescan, S. Le Bras, V. H. Lefebvre, U. Frandsen, T. Klein, M. Foschi, D. G. Pipeleers, R. Scharfmann, O. D. Madsen, H. Heimberg, *Lab Invest* **2005**, *85* (1), 65.

[18] X. Fang, D. Chen, X. Yang, X. Cao, Q. Cheng, K. Liu, P. Xu, Y. Wang, J. Xu, S. Zhao, Z. Yan, *Cell Death Discov* **2024**, *10* (1), 111.

[19] C. J. Hanley, S. Waise, M. J. Ellis, M. A. Lopez, W. Y. Pun, J. Taylor, R. Parker, L. M. Kimbley, S. J. Chee, E. C. Shaw, J. West, A. Alzetani, E. Woo, C. H. Ottensmeier, M. J. J. Rose-Zerilli, G. J. Thomas, *Nat Commun* **2023**, *14* (1), 387.

[20] J. C. Schupp, T. S. Adams, C. Cosme, Jr., M. S. B. Raredon, Y. Yuan, N. Omote, S. Poli, M. Chioccioli, K. A. Rose, E. P. Manning, M. Sauler, G. DeIuliis, F. Ahangari, N. Neumark, A. C. Habermann, A. J. Gutierrez, L. T. Bui, R. Lafyatis, R. W. Pierce, K. B. Meyer, M. C. Nawijn, S. A. Teichmann, N. E. Banovich, J. A. Kropski, L. E. Niklason, D. Pe'er, X. Yan, R. J. Homer, I. O. Rosas, N. Kaminski, *Circulation* **2021**, *144* (4), 286.

[21] C. Tacconi, Y. He, L. Ducoli, M. Detmar, *Angiogenesis* **2021**, *24* (1), 67.

[22] A. F. Møller, J. G. S. Madsen, *Nat Commun* **2023**, *14* (1), 8473.

[23] J. Cao, D. R. O'Day, H. A. Pliner, P. D. Kingsley, M. Deng, R. M. Daza, M. A. Zager, K. A. Aldinger, R. Blecher-Gonen, F. Zhang, M. Spielmann, J. Palis, D. Doherty, F. J. Steemers, I. A. Glass, C. Trapnell, J. Shendure, *Science* **2020**, *370* (6518).

[24] a) J. M. Baer, C. Zuo, L. I. Kang, A. A. de la Lastra, N. C. Borcherding, B. L. Knolhoff, S. J. Bogner, Y. Zhu, L. Yang, J. Laurent, M. A. Lewis, N. Zhang, K. W. Kim, R. C. Fields, W. M. Yokoyama, J. C. Mills, L. Ding, G. J. Randolph, D. G. DeNardo, *Nat Immunol* **2023**, *24* (9), 1443; b) J. Li, T. Wei, K. Ma, J. Zhang, J. Lu, J. Zhao, J. Huang, T. Zeng, Y. Xie, Y. Liang, X. Li, Q. Zhang, T. Liang, *Cancer Lett* **2024**, *584*, 216607.

[25] O. E. Olaniru, U. Kadolsky, S. Kannambath, H. Vaikkinen, K. Fung, P. Dhami, S. J. Persaud, *Cell Metab* **2023**, *35* (1), 184.

[26] R. Bohuslavova, V. Fabriciova, O. Smolik, L. Lebrón-Mora, P. Abaffy, S. Benesova, D. Zucha, L. Valihrach, Z. Berkova, F. Saudek, G. Pavlinkova, *Nat Commun* **2023**, *14* (1), 5554.

[27] S. E. Di Carlo, J. Raffenne, H. Varet, A. Ode, D. C. Granados, M. Stein, R. Legendre, J. Tuckermann, C. Bousquet, L. Peduto, *Nat Immunol* **2023**, *24* (11), 1867.

[28] S. A. Sarkar, S. Kobberup, R. Wong, A. D. Lopez, N. Quayum, T. Still, A. Kutchma, J. N. Jensen, R. Gianani, G. M. Beattie, J. Jensen, A. Hayek, J. C. Hutton, *Diabetologia* **2008**, *51* (2), 285.

[29] J. Thorlacius-Ussing, C. Jensen, N. I. Nissen, T. R. Cox, R. Kalluri, M. Karsdal, N. Willumsen, *J Pathol* **2024**, *262* (1), 22.

[30] D. Zhang, M. X. Sliwkowski, M. Mark, G. Frantz, R. Akita, Y. Sun, K. Hillan, C. Crowley, J. Brush, P. J. Godowski, *Proc Natl Acad Sci U S A* **1997**, *94* (18), 9562.

[31] B. Wu, S. Wei, N. Petersen, Y. Ali, X. Wang, T. Bacaj, P. Rorsman, W. Hong, T. C. Südhof, W. Han, *Proc Natl Acad Sci U S A* **2015**, *112* (32), 9996.

[32] R. Hidaka, M. Machida, S. Fujimaki, K. Terashima, M. Asashima, T. Kuwabara, *Stem Cell Res Ther* **2013**, *4* (3), 51.

[33] A. Silva, A. R. M. Almeida, A. Cachucho, J. L. Neto, S. Demeyer, M. de Matos, T. Hogan, Y. Li, J. Meijerink, J. Cools, A. R. Grosso, B. Seddon, J. T. Barata, *Blood* **2021**, *138* (12), 1040.

[34] T. Zhou, W. Damsky, O. E. Weizman, M. K. McGeary, K. P. Hartmann, C. E. Rosen, S. Fischer, R. Jackson, R. A. Flavell, J. Wang, M. F. Sanmamed, M. W. Bosenberg, A. M. Ring, *Nature* **2020**, *583* (7817), 609.

[35] E. Park, W. E. Barclay, A. Barrera, T. C. Liao, H. R. Salzler, T. E. Reddy, M. L. Shinohara, M. Ciofani, *Sci Immunol* **2023**, *8* (88), eadg7597.

[36] K. Motomura, T. Matsuzaka, S. Shichino, T. Ogawa, H. Pan, T. Nakajima, Y. Asano, T. Okayama, T. Takeuchi, H. Ohno, S. I. Han, T. Miyamoto, Y. Takeuchi, M. Sekiya, H. Sone, N. Yahagi, Y. Nakagawa, T. Oda, S. Ueha, K. Ikeo, A. Ogura, K. Matsushima, H. Shimano, *Diabetes* **2024**, *73* (1), 75.

[37] a) Y. Jin, L. Muhl, M. Burmakin, Y. Wang, A. C. Duchez, C. Betsholtz, H. M. Arthur, L. Jakobsson, *Nat Cell Biol* **2017**, *19* (6), 639; b) E. Trimm, K. Red-Horse, *Nat Rev Cardiol* **2023**, *20* (3), 197.

[38] A. Egozi, K. Bahar Halpern, L. Farack, H. Rotem, S. Itzkovitz, *Cell Rep* **2020**, *32* (7), 108043.

[39] T. Athwal, W. Huang, R. Mukherjee, D. Latawiec, M. Chvanov, R. Clarke, K. Smith, F. Campbell, C. Merriman, D. Criddle, R. Sutton, J. Neoptolemos, N. Vlatković, *Cell Death Dis* **2014**, *5* (4), e1165.

[40] A. L. Li, K. Sugiura, N. Nishiwaki, K. Suzuki, D. Sadeghian, J. Zhao, A. Maitra, D. Falvo, R. Chandwani, J. R. Pitarresi, P. A. Sims, A. K. Rustgi, *Dev Cell* **2024**, *59* (22), 3025.

[41] M. E. Sabbatini, X. Chen, S. A. Ernst, J. A. Williams, *J Biol Chem* **2008**, *283* (35), 23884.

[42] R. Tabassum, G. Chauhan, O. P. Dwivedi, A. Mahajan, A. Jaiswal, I. Kaur, K. Bandesh, T. Singh, B. J. Mathai, Y. Pandey, M. Chidambaram, A. Sharma, S. Chavali, S. Sengupta, L. Ramakrishnan, P. Venkatesh, S. K. Aggarwal, S. Ghosh, D. Prabhakaran, R. K. Srinath, M. Saxena, M. Banerjee, S. Mathur, A. Bhansali, V. N. Shah, S. V. Madhu, R. K. Marwaha, A. Basu, V. Scaria, M. I. McCarthy, R. Venkatesan, V. Mohan, N. Tandon, D. Bharadwaj, *Diabetes* **2013**, *62* (3), 977.

[43] J. Martinez-Pinna, L. Marroqui, A. Hmadcha, J. Lopez-Beas, S. Soriano, S. Villar-Pazos, P. Alonso-Magdalena, R. S. Dos Santos, I. Quesada, F. Martin, B. Soria, J. Gustafsson, A. Nadal, *Diabetologia* **2019**, *62* (9), 1667.

[44] N. Aldous, A. K. Elsayed, B. Memon, S. Ijaz, S. Hayat, E. M. Abdelalim, *Diabetologia* **2024**, *67* (12), 2786.

[45] M. Z. Zhang, W. Mai, C. Li, S. Y. Cho, C. Hao, G. Moeckel, R. Zhao, I. Kim, J. Wang, H. Xiong, H. Wang, Y. Sato, Y. Wu, Y. Nakanuma, M. Lilova, Y. Pei, R. C. Harris, S. Li, R. J. Coffey, L. Sun, D. Wu, X. Z. Chen, M. D. Breyer, Z. J. Zhao, J. A. McKanna, G. Wu, *Proc Natl Acad Sci U S A* **2004**, *101* (8), 2311.

[46] L. W. van Laake, S. van den Driesche, S. Post, A. Feijen, M. A. Jansen, M. H. Driessens, J. J. Mager, R. J. Snijder, C. J. Westermann, P. A. Doevendans, C. J. van Echteld, P. ten Dijke, H. M. Arthur, M. J. Goumans, F. Lebrin, C. L. Mummery, *Circulation* **2006**, *114* (21), 2288.

[47] M. Bobowski-Gerard, C. Boulet, F. P. Zummo, J. Dubois-Chevalier, C. Gheeraert, M. Bou Saleh, J. M. Strub, A. Farce, M. Ploton, L. Guille, J. Vandel, A. Bongiovanni, N. Very, E. Woitrain, A. Deprince, F. Lalloyer, E. Bauge, L. Ferri, L. C. Ntandja-Wandji, A. K. Cotte, C. Grangette, E. Vallez, S. Cianférani, V. Raverdy, R. Caiazzo, V. Gnemmi, E. Leteurtre, B. Pourcet, R. Paumelle, K. Ravnskjaer, G. Lassailly, J. T. Haas, P. Mathurin, F. Pattou, L. Dubuquoy, B. Staels, P. Lefebvre, J. Eeckhoute, *Nat Commun* **2022**, *13* (1), 5324.

[48] T. R. Hammond, C. Dufort, L. Dissing-Olesen, S. Giera, A. Young, A. Wysoker, A. J. Walker, F. Gergits, M. Segel, J. Nemesh, S. E. Marsh, A. Saunders, E. Macosko, F. Ginhoux, J. Chen, R. J. M. Franklin, X. Piao, S. A. McCarroll, B. Stevens, *Immunity* **2019**, *50* (1), 253.

[49] M. Porcu, M. Kleppe, V. Gianfelici, E. Geerdens, K. De Keersmaecker, M. Tartaglia, R. Foà, J. Soulier, B. Cauwelier, A. Uyttebroeck, E. Macintyre, P. Vandenberghe, V. Asnafi, J. Cools, *Blood* **2012**, *119* (19), 4476.

[50] V. Secq, J. Leca, C. Bressy, F. Guillaumond, P. Skrobuk, J. Nigri, S. Lac, M. N. Lavaut, T. T. Bui, A. K. Thakur, N. Callizot, R. Steinschneider, P. Berthezene, N. Dusetti, M. Ouaissi, V. Moutardier, E. Calvo, C. Bousquet, S. Garcia, G. Bidaut, S. Vasseur, J. L. Iovanna, R. Tomasini, *Cell Death Dis* **2015**, *6* (1), e1592.

[51] M. E. Vail, C. Murone, A. Tan, L. Hii, D. Abebe, P. W. Janes, F. T. Lee, M. Baer, V. Palath, C. Bebbington, G. Yarranton, C. Llerena, S. Garic, D. Abramson, G. Cartwright, A. M. Scott, M. Lackmann, *Cancer Res* **2014**, *74* (16), 4470.

[52] Y. Lee, D. Bogdanoff, Y. Wang, G. C. Hartoularos, J. M. Woo, C. T. Mowery, H. M. Nisonoff, D. S. Lee, Y. Sun, J. Lee, S. Mehdizadeh, J. Cantlon, E. Shifrut, D. N. Ngyuen, T. L. Roth, Y. S. Song, A. Marson, E. D. Chow, C. J. Ye, *Sci Adv* **2021**, *7* (17).

[53] Y. Fu, M. Zhang, B. Sui, F. Yuan, W. Zhang, Y. Weng, L. Xiang, C. Li, L. Shao, Y. You, X. Mao, H. Zeng, D. Chen, M. Zhang, S. Shi, X. Hu, *Theranostics* **2024**, *14* (8), 3385.

[54] L. Uusküla-Reimand, M. D. Wilson, *Sci Adv* **2022**, *8* (44), eadd4920.

[55] J. Cao, D. A. Cusanovich, V. Ramani, D. Aghamirzaie, H. A. Pliner, A. J. Hill, R. M. Daza, J. L. McFaline-Figueroa, J. S. Packer, L. Christiansen, F. J. Steemers, A. C. Adey, C. Trapnell, J. Shendure, *Science* **2018**, *361* (6409), 1380.

[56] A. K. Y. Yim, P. L. Wang, J. R. Bermingham, Jr., A. Hackett, A. Strickland, T. M. Miller, C. Ly, R. D. Mitra, J. Milbrandt, *Nat Neurosci* **2022**, *25* (2), 238.
